# Human pluripotent stem cell-derived kidney organoids for personalized congenital and idiopathic nephrotic syndrome modeling

**DOI:** 10.1242/dev.200198

**Published:** 2022-05-06

**Authors:** Jitske Jansen, Bartholomeus T. van den Berge, Martijn van den Broek, Rutger J. Maas, Deniz Daviran, Brigith Willemsen, Rona Roverts, Marit van der Kruit, Christoph Kuppe, Katharina C. Reimer, Gianluca Di Giovanni, Fieke Mooren, Quincy Nlandu, Helmer Mudde, Roy Wetzels, Dirk den Braanker, Naomi Parr, James S. Nagai, Vedran Drenic, Ivan G. Costa, Eric Steenbergen, Tom Nijenhuis, Henry Dijkman, Nicole Endlich, Nicole C. A. J. van de Kar, Rebekka K. Schneider, Jack F. M. Wetzels, Anat Akiva, Johan van der Vlag, Rafael Kramann, Michiel F. Schreuder, Bart Smeets

**Affiliations:** 1 Department of Pathology, Radboud Institute for Molecular Life Sciences, Radboud University Medical Center, PO Box 9101, 6500 HB Nijmegen, The Netherlands; 2 Department of Pediatric Nephrology, Radboud Institute for Molecular Life Sciences, Radboud University Medical Center, Amalia Children's Hospital, PO Box 9101, 6500 HB Nijmegen, The Netherlands; 3 Division of Nephrology and Clinical Immunology, Institute of Experimental Medicine and Systems Biology, Medical Faculty RWTH Aachen University, Pauwelsstrasse 30, 52074 Aachen, Germany; 4 Department of Nephrology, Radboud Institute for Molecular Life Sciences, Radboud University Medical Center, PO Box 9101, 6500 HB Nijmegen, The Netherlands; 5 Department of Biochemistry, Electron Microscopy Center, Radboudumc Technology Center Microscopy, Radboud Institute of Molecular Life Sciences, Radboud University Medical Center, Geert Grooteplein 29, 6525 GA Nijmegen, The Netherlands; 6 Division of Nephrology and Clinical Immunology, RWTH Aachen University, Aachen 52062, Germany; 7 Institute for Biomedical Technologies, Department of Cell Biology, RWTH Aachen University, Aachen 52062, Germany; 8 Institute for Computational Genomics, University Hospital RWTH Aachen, Achen 52062, Germany; 9 Joint Research Center for Computational Biomedicine, RWTH Aachen University Hospital, Aachen 52062, Germany; 10 NIPOKA, 17489 Greifswald, Germany; 11 Department of Anatomy and Cell Biology, University Medicine Greifswald, 17489 Greifswald, Germany; 12 Department of Developmental Biology, Erasmus Medical Center, Rotterdam 3015 GD, The Netherlands; 13 Oncode Institute, Erasmus Medical Center, Rotterdam, The Netherlands; 14 Department of Internal Medicine, Nephrology and Transplantation, Erasmus Medical Center, Rotterdam 3015 GD, The Netherlands

**Keywords:** Nephrotic syndrome, Human iPSC-derived kidney organoids, 2D iPSC-derived podocytes

## Abstract

Nephrotic syndrome (NS) is characterized by severe proteinuria as a consequence of kidney glomerular injury due to podocyte damage. *In vitro* models mimicking *in vivo* podocyte characteristics are a prerequisite to resolve NS pathogenesis. The detailed characterization of organoid podocytes resulting from a hybrid culture protocol showed a podocyte population that resembles adult podocytes and was superior compared with 2D counterparts, based on single-cell RNA sequencing, super-resolution imaging and electron microscopy. In this study, these next-generation podocytes in kidney organoids enabled personalized idiopathic nephrotic syndrome modeling, as shown by activated slit diaphragm signaling and podocyte injury following protamine sulfate, puromycin aminonucleoside treatment and exposure to NS plasma containing pathogenic permeability factors. Organoids cultured from cells of a patient with heterozygous *NPHS2* mutations showed poor *NPHS2* expression and aberrant *NPHS1* localization, which was reversible after genetic correction. Repaired organoids displayed increased VEGFA pathway activity and transcription factor activity known to be essential for podocyte physiology, as shown by RNA sequencing. This study shows that organoids are the preferred model of choice to study idiopathic and congenital podocytopathies.

## Abstract (precis)

**Summary:** Kidney organoid podocytes generated from human pluripotent stem cells using a hybrid differentiation protocol allow podocyte pathophysiology modeling that leads to congenital as well as idiopathic nephrotic syndrome in patients.

## INTRODUCTION

Podocytes play an important role in the glomerular filtration barrier. Podocytes possess interdigitating foot processes that are bridged by a protein complex called the slit diaphragm, which contains proteins such as nephrin (*NPHS1*) and podocin (*NPHS2*). Malfunctioning of podocytes (podocytopathy) causes massive proteinuria, resulting in nephrotic syndrome (NS) (Shabaka et al., 2020; Veissi et al., 2020). In patients with idiopathic NS (iNS) a kidney biopsy shows no abnormalities under light microscopy. Complete foot process effacement, as determined by electron microscopy, defines iNS as a podocytopathy. The identification of mutations in congenital nephrotic syndrome in important podocyte genes (*NPHS1* and *NPHS2*) (Maas et al., 2016; Noone et al., 2018) also defines iNS as a podocytopathy.

The pathophysiology of idiopathic NS has not yet been clarified. Within the spectrum of NS, a unique subset of patients have presumed pathogenic circulating permeability factors (CPFs) that result in podocyte injury and eventually recurrence of the disease after kidney transplantation. Clinical evidence suggests that CPFs involve the immune system (i.e. T and B cells) (Colucci et al., 2018; Gallon et al., 2012; Straatmann et al., 2014). However, there is still no consensus about the nature of the CPFs (Maas et al., 2014; Rudnicki, 2016; Shoji et al., 2020).

In order to gain more insight into podocytopathies, animal models and basic *in vitro* models have been explored, but these have failed to provide full mechanistic insight. Hence, there is an unmet need for models that recapitulate glomerular physiology and pathology to resolve the molecular mechanism of underlying glomerular diseases. Previously, podocyte cell lines, human induced pluripotent stem cell (iPSC)-derived 2D podocytes, and 3D kidney organoid models have been established (Garreta et al., 2019; Musah et al., 2017; Rauch et al., 2018; Saleem et al., 2002; Sharmin et al., 2016; Takasato et al., 2015; Wu et al., 2018). The advantages of 2D podocyte models are the straightforward and standardized culture conditions, although the presence and localization of slit diaphragm proteins are not convincing (Hale et al., 2018; van den Broek et al., 2021). Kidney organoid models are more complex and require challenging culture protocols, but podocyte characteristics, including slit diaphragm proteins, have been demonstrated (Hale et al., 2018; Taguchi and Nishinakamura, 2017; van den Berg et al., 2018; Wu et al., 2018). Despite the substantial progress that has been made in the kidney organoid field, current organoids resemble immature tissue, show limited vascularization, and do not have a functional glomerular filtration barrier (Takasato et al., 2015; Uchimura et al., 2020; van den Berg et al., 2018). Recently, Tanigawa and colleagues successfully modeled Finnish-type congenital NS (*NPHS1* mutant) in kidney organoids, identified slit diaphragm abnormalities in these podocytes and could correct the mutation genetically (Tanigawa et al., 2018). The study by Tanigawa et al. emphasized the potential of kidney organoids for personalized regenerative medicine and for the accurate investigation of NS *in vitro*.

The aim of our study was to investigate the potency of current kidney organoids to model podocytopathies, as observed in NS, accurately. In this study, we established a hybrid differentiation protocol that resulted in kidney organoids containing a podocyte population that approaches adult podocyte expression levels. We succeeded in modeling podocyte pathophysiology that leads to congenital (*NPHS2* mutant) NS as well as idiopathic NS in patients. Our data indicate that iPSC-derived kidney organoids are the preferred *in vitro* system to model NS, because cellular signaling cascades are induced upon injury that are absent in 2D iPSC-derived podocytes and a podocyte cell line.

## RESULTS

### Podocytes in kidney organoids show superior characteristics compared with 2D podocytes

**Fig. 1.**
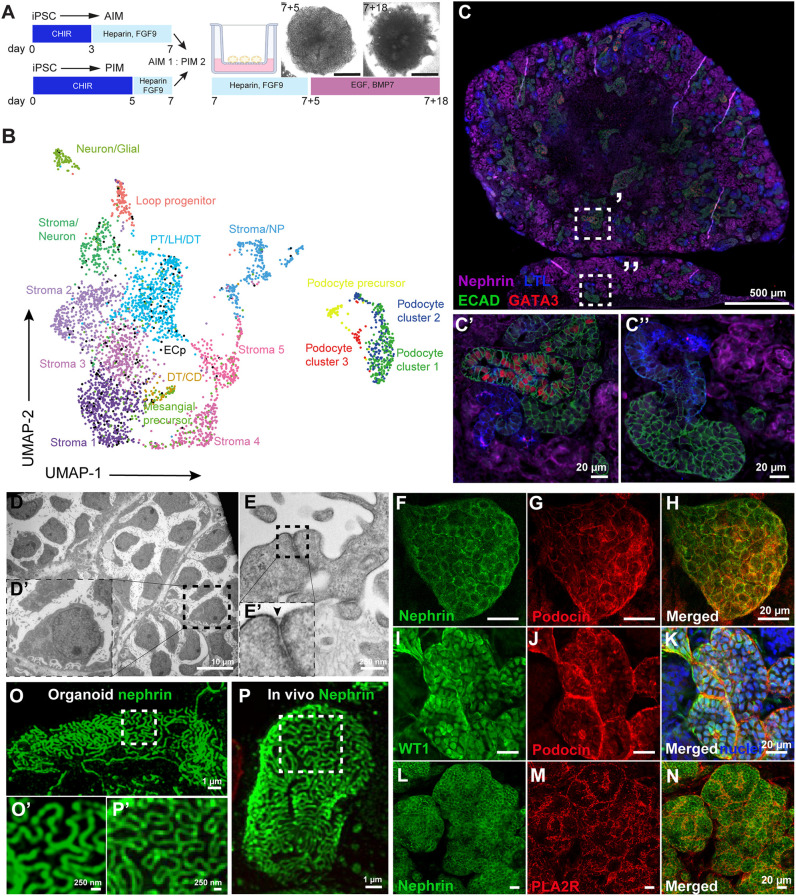
**Human iPSC-derived kidney organoids contain 17 distinct cell clusters, including well-developed podocytes showing filtration slits.** (A) Schematic of the iPSC-derived kidney organoid culture protocol. iPSCs were differentiated in 2D either towards anterior intermediate mesoderm (AIM) or posterior intermediate mesoderm (PIM) and aggregates (300,000 cells) were made using a 1:2 AIM/PIM ratio on day 7. Organoids were cultured on the air-liquid interface of Transwell™ filters for an additional 18 days, using a mixture of growth factors as indicated. BMP7, bone morphogenic protein 7; CHIR, CHIR 99021; EGF, epidermal growth factor; FGF9, fibroblast growth factor 9. (B) Uniform manifold approximation and projection (UMAP) integration of 3900 single cells from human iPSC-derived kidney organoids results in 17 different cell clusters. CD, collecting duct precursors; DT, distal tubular cells; ECp, endothelial cell precursors; LH, Loop of Henle cells; NP, neuronal progenitors; PT, proximal tubular cells. (C-C″) Segmented patterning in human kidney organoids shows the presence of podocytes (NPHS1, magenta), proximal tubules [lotus tetragonolobus lectin (LTL), blue], distal-like [E-cadherin (ECAD), green] and collecting duct-like [ECAD (green) and GATA binding protein 3 (GATA3) (red)] cells. (D-E′) TEM micrographs of organoid-derived podocytes showing cell bodies and interdigitating foot processes including a filtration slit, indicated by an arrowhead in E′. (F-H) Filtration slit proteins podocin (red) and nephrin (green) are expressed at the podocyte cell membranes. (I-K) The podocyte-specific transcription factor Wilms' Tumor 1 (WT1, green) is expressed in podocyte nuclei (podocin, red; nuclei, blue). (L-N) Phospholipase A2 receptor (PLA2R, red) expression at the podocyte cell membranes (nephrin, green). (O-P′) This cluster was subsetted and merged with the subsetted podocytes from the organoids. Super-resolution filtration slit imaging of nephrin in organoid-podocytes (O,O′) and in a morphologically healthy appearing glomerulus of a Munich Wistar Frömter rat (P,P′).

Human iPSCs were successfully cultured into kidney organoids using a hybrid directed differentiation protocol based on that described by Uchimura and Takasato et al. (Takasato et al., 2015; Uchimura et al., 2020). Briefly, iPSCs were differentiated towards metanephric mesenchyme (MM) by exposure to the WNT signaling agonist CHIR 99021 for 5 days and into GATA3^+^ epithelium by CHIR 99021 exposure for 3 days (Takasato et al., 2015). On day 7, GATA3^+^ and MM cells were mixed in a 1:2 ratio, to simulate signaling cues between both populations that are essential for nephrogenesis (Fig. 1A) (Uchimura et al., 2020). Using this approach, segmented patterning was induced and organoids consisted of kidney cells, including podocytes, proximal tubule, loop of Henle, distal tubule, and collecting duct-like cells, as well as endothelial and mesangial cell precursors, stromal cells, and off-target neuronal progenitors, as shown by single-cell RNA sequencing (scRNA-seq) and immunofluorescence staining (Fig. 1B-C″, Fig. S1, Table S1).

Podocytes that mirror *in vivo* characteristics, e.g. the presence of slit diaphragm proteins, as closely as possible are required to study NS *in vitro*. Hence, the podocytes in the kidney organoids were thoroughly characterized. The presence of podocyte cell bodies and foot processes as well as a slit diaphragm could be observed at the ultrastructural level (Fig. 1D-E′). Podocyte-specific proteins, such as podocin, nephrin, Wilms' tumor-1 (WT1), and synaptopodin were also expressed (Fig. 1F-K, Fig. S2). Phospholipase A2 receptor (PLA2R; also known as PLA2R1) was also detected in organoid podocytes (Fig. 1L-N). PLA2R is the main target receptor of autoantibodies present in the majority of NS patients with primary membranous nephropathy (van de Logt et al., 2019). Using super-resolution imaging, slit diaphragms that showed nephrin expression could be detected between podocyte foot processes in organoids, showing similar morphological characteristics to *in vivo* adult slit diaphragms (Fig. 1O-P′).

**Fig. 2.**
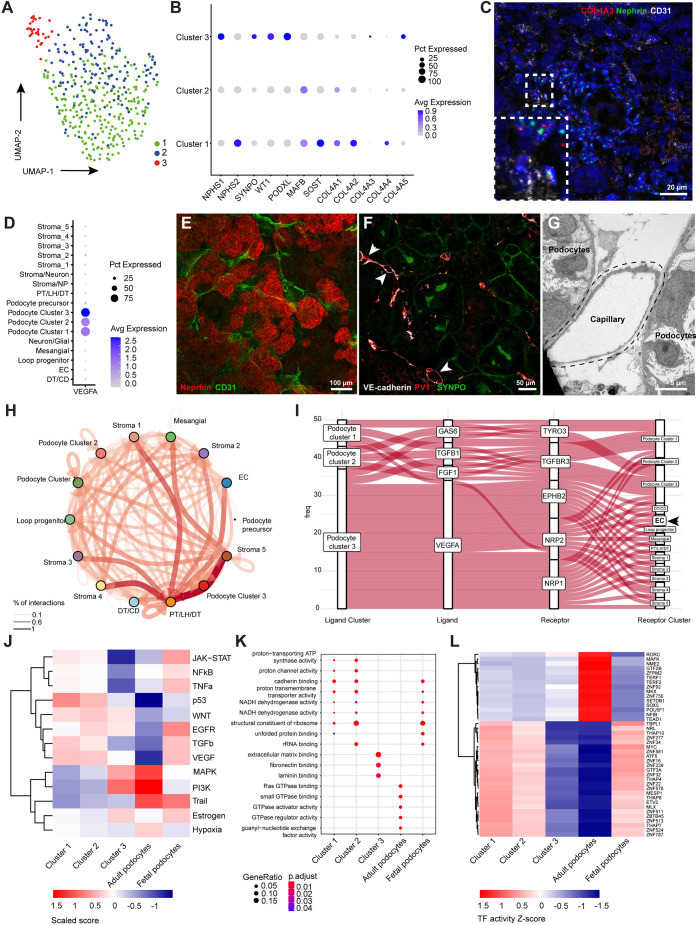
**Kidney organoid-derived podocyte subcluster 3 partially resembles adult podocytes.** (A,B) UMAP integration of 401 podocytes (A) identified three subclusters based on differentially expressed genes (B). Avg, average; Pct, percent. (C) RNAscope analysis showing nephrin (NPHS1), collagen IV alpha three (COL4A3) and endothelial marker cluster of differentiation 31 (CD31) expression. Inset shows an enlarged view of the boxed area. (D) Vascular endothelial growth factor A (VEGFA) expression among the podocyte subclusters. (E,F) CD31-, VE-cadherin- and plasmalemmal vesicle associated protein-1 (PV-1)-stained endothelial capillaries are interspaced between podocytes expressing NPHS1 (E) and synaptopodin (SYNPO) (F) in the organoids. Arrowheads indicate endothelial capillaries with lumen. (G) Ultrastructural analysis showing the luminal space of a capillary adjacent to organoid podocytes. (H) Cell-cell interactions between all cells present in kidney organoids using CellPhoneDB and CrossTalkeR packages. Thickness and depth of color of the arrows correspond to the percentage of interactions. (I) Autocrine and paracrine podocyte ligand-receptor interactions showing a dominant role for VEGFA signaling originating from cluster 3 to VEGF-related receptors in, amongst others, endothelial cell precursors (EC). Analysis was performed using CellPhoneDB and CrossTalkeR packages. (J-L) Comparative PROGENy (J), GO terms (K) and DoRothEA analysis (L) showing pathway activity (J), biological processes (K) and transcription factor activity (L) between the three podocyte subclusters, adult and fetal podocytes [adult and fetal podocytes sequencing data extracted from Gene Expression Omnibus (GSE118184 and GSE112570)]. Abbreviations as in Fig. 1.

Analysis of scRNA-seq data revealed three podocyte subclusters, each expressing a distinct profile of podocyte markers, independent of cell cycle phase (Fig. 2A,B, Fig. S3A). Notably, cluster 3 showed expression of the collagen 4 alpha 3, 4 and 5 (*COL4A3*, *COL4A4* and *COL4A5*) genes (Fig. 2B, Fig. S3B). The developmental expression of the aforementioned collagen chains is initiated at the capillary loop stage in fetal podocytes, whereas expression is exclusive for mature podocytes (Abrahamson et al., 2009) (Fig. S3B). The *COL4A3* mRNA expression was confirmed in organoid podocytes (*NPHS1*^+^) using *in situ* hybridization (Fig. 2C). During nephrogenesis, at the S-shaped body phase, podocytes start to secrete vascular endothelial growth factor A (VEGFA), which is essential for podocyte differentiation and attraction of endothelial cell precursors, as a first step towards the formation of a glomerular filtration barrier (Abrahamson, 2012; Eremina et al., 2003). All organoid podocyte subclusters expressed VEGFA, with cluster 3 showing the most prominent VEGFA expression (Fig. 2D). In fact, VEGFA expression in cluster 3 was 1.5- to 2-fold higher than the expression observed in fetal or adult control podocytes (Fig. S3C). The interaction between podocytes and endothelial cells dictates glomerular filtration barrier formation and is essential for glomerular maturation (Schell et al., 2014; Sison et al., 2010), which, notably, was not observed in our kidney organoids. Cluster of differentiation 31 (CD31; PECAM1), vascular endothelial (VE)-cadherin (CDH5), plasmalemmal vesicle associated protein-1 (PV-1; PLVAP) and Eps15 homology domain-containing 3 (EHD3) were expressed by endothelial capillary structures (Fig. 2C,E-G, Fig. S4), including luminal spaces in the organoids. However, organoid vasculature was interspaced between podocytes rather than invading as normally occurs at the capillary loop stage during glomerulogenesis *in vivo*.

To understand the observed maturation differences within the three clusters of podocytes, we further investigated the cell-cell communication between podocytes and endothelial cells by performing ligand-receptor analysis using CellPhoneDB and CrossTalkeR packages (Fig. 2H) (Efremova et al., 2020; Nagai et al., 2021). Podocyte cluster 3 showed enriched VEGFA interactions with the endothelial cell precursor receptors ephrin type-B receptor 2 (EPHB2), neuropilin 1 and neuropilin 2 (NRP1 and -2), which could not be identified in the other podocyte clusters (Fig. 2I). Concomitantly, using PROGENy (Schubert et al., 2018), podocyte cluster 3 showed an increased phosphoinositide 3-kinase (PI3K) pathway activity (Fig. 2J) which, in concert with VEGF signaling, is associated with *COL4A3* gene regulation (Chen et al., 2004; Veron et al., 2012). Podocyte cluster 3 mimics adult podocytes most closely when compared with fetal and adult pathway activities that were extracted from the Kidney Interactive Transcriptomics database (Fig. 2J) (The Humphreys Laboratory; https://humphreyslab.com/SingleCell/). Gene ontology (GO) terms showed distinct biological processes related to extracellular matrix binding in podocyte cluster 3, which were not significantly enriched in the other podocyte clusters or in adult podocytes (Fig. 2K). GO terms of podocyte cluster 1 and 2 showed overlap with biological processes active in fetal podocytes (Fig. 2K). To infer transcription factor (TF) activity differences between the podocyte clusters that may aid in resolving molecular cues involved in podocyte maturation, we used the DoRothEA computational package (Garcia-Alonso et al., 2019). We found that podocyte clusters 1 and 2 resemble a fetal podocyte TF profile, whereas the transcriptional circuit in podocyte cluster 3 showed partial overlap with adult podocytes (Fig. 2L). However, some emerging TFs, such as TEA domain transcription factor 1 (TEAD1) and nuclear factor I B (NFIB), involved in cellular differentiation and tissue homeostasis, were less active in podocyte cluster 3 compared with adult podocytes (Fig. 2L) (Adam et al., 2020; Rinschen et al., 2017; Sabò et al., 2014). Of note, zinc finger protein 750 (ZNF750) was less enriched in cluster 3 podocytes compared to adult podocytes. Normally, ZNF750 represses kruppel like factor 4 (KLF4), a known epigenetic modulator involved in podocyte differentiation (Hayashi et al., 2014). Also, SRY-Box transcription factor 5 (SOX5), known to co-activate WT1 (Dong et al., 2015), shows limited activity in cluster 3. Altogether, we show that essential podocyte markers are expressed in the entire kidney organoid podocyte population, but we identified a subpopulation of podocytes that resemble adult podocytes at the transcriptional level.

**Fig. 3.**
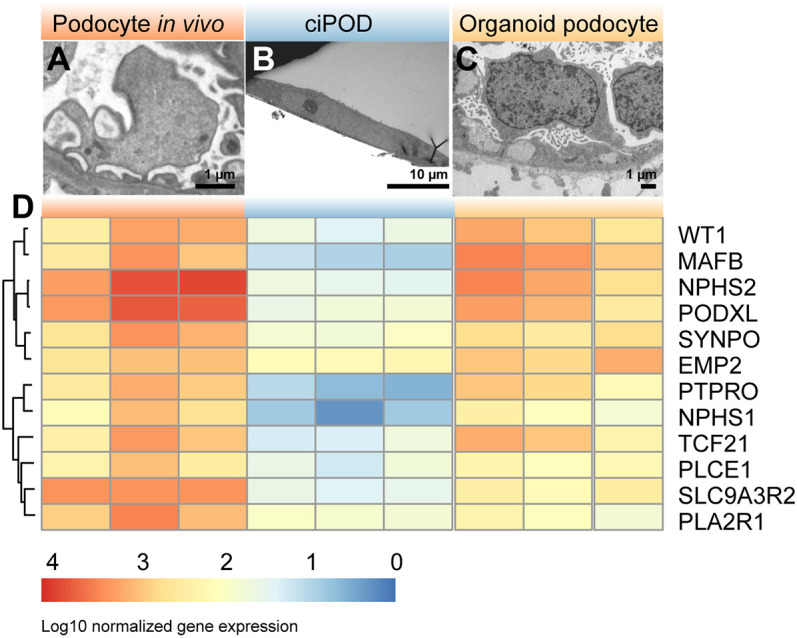
**Organoid podocytes show superior morphology and transcriptome compared with 2D podocytes *in vitro*.** (A-C) TEM micrographs showing the ultrastructural analysis of normal human podocytes *in situ* (A), conditionally immortalized podocytes (ciPOD) (B) and organoid podocytes (C). (D) Heatmap of bulk RNA sequencing data showing the expression signature of podocyte-specific markers in normal human glomeruli, ciPODs and organoid podocytes.

Next, we compared the performance of the current organoid model with a frequently used human 2D *in vitro* podocyte cell line, i.e. a widely used conditionally immortalized podocyte cell line (ciPOD) (Saleem et al., 2002). Morphologically, ciPODs do not show the typical appearance of a ‘floating’ cell body with primary and secondary (foot process) structures (Fig. 3B). Notably, within the organoids, podocytes strongly resembled human podocytes (Figs 1E′ and 3A-C). Transcriptome analysis revealed that organoid podocytes resemble human podocytes much more than ciPODs do (Fig. 3D), in particular when evaluating typical podocyte markers such as *NPHS1*, *NPHS2*, *WT1*, *PODXL* and *SYNPO*. Altogether, the organoid podocytes in part resemble the transcriptome profile of human adult kidney podocytes, which includes the expression of typical podocyte markers that are essential for the study of NS pathology *in vitro*.

### Impaired protein processing towards the cell membrane in kidney organoids of a patient with *NPHS2* mutations is abrogated after genetic correction

Podocin is known to play an important role in slit diaphragm organization. Intact podocin is required for nephrin membrane trafficking, ultimately leading to oligomerization of podocin clusters and nephrin, which is required for development of filtration slits. NS patients with mutated *NPHS2* exhibit aberrant nephrin expression, thereby emphasizing the crucial role of podocin in podocyte physiology (Jaffer et al., 2011; Zhang et al., 2004). Here, we used kidney organoids to model the congenital nephrotic syndrome caused by *NPHS2* mutation.

**Fig. 4.**
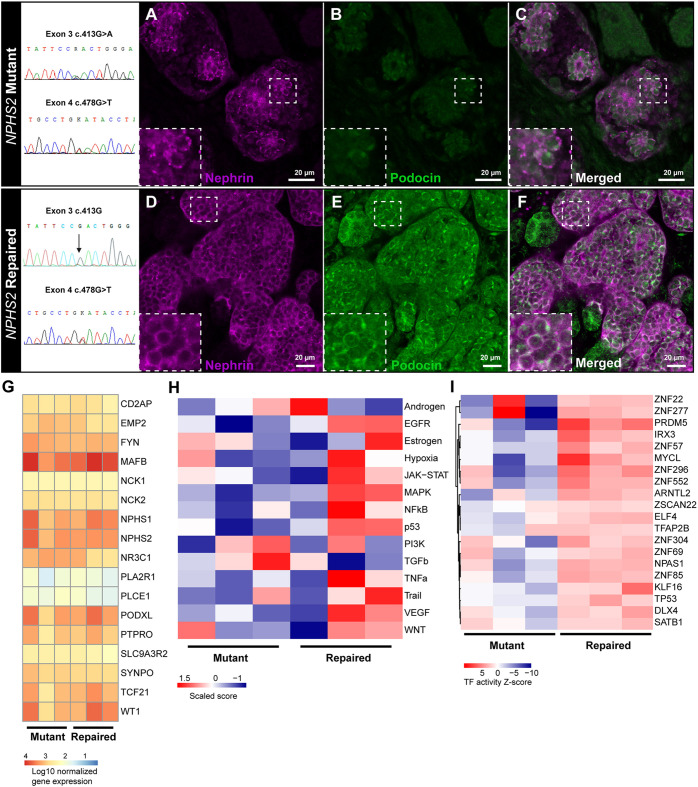
**Congenital nephrotic syndrome modeling of patient-derived *NPHS2* mutant and genetically repaired iPSC kidney organoids.** (A-F) Nephrin (magenta) and podocin (green) expression in *NPHS2* mutant (A-C) and genetically repaired (D-F) kidney organoids. Insets show enlarged views of the boxed areas. (G) Comparative bulk transcriptomics of podocyte-specific genes in mutant versus repaired organoid podocytes. (H,I) Comparative PROGENy pathway activity analysis (H) and DoRothEA transcription factor activity (I) of mutant versus repaired organoid podocytes. (G-I) Bulk transcriptomics was performed from RNA isolated from three independent batches of NPHS1^+^ sorted podocytes from mutant and repaired organoids. Per batch, ten mutant or repaired organoids were pooled and used for sorting podocytes by FACS.

Compound heterozygous mutations p.Arg138Gln (exon 3) and p.Asp160Tyr (exon 4) in the podocin (*NPHS2*) gene were identified in a patient with congenital nephrotic syndrome. Erythroblasts from this patient were successfully reprogrammed into iPSCs and cultured into organoids. Podocin protein expression was almost absent on the cell membrane and, although nephrin expression was present, membrane expression was patchy or in some podocytes a weak cytoplasmic stain was detected (Fig. 4A-C), which is in line with observations in NS patients (Zhang et al., 2004). Correction of the exon 3 mutation by CRISPR/Cas9 resulted in restored podocin protein expression colocalizing with nephrin expression at the cell membrane in organoids (Fig. 4D-F). Bulk transcriptomics of organoid *NPHS1*^+^ podocytes showed a distinct podocyte mRNA expression profile for both mutant and repaired organoid podocytes, for example *SYNPO*, *MAFB*, *WT1*, *NPHS1* and *NPHS2* expression (Fig. 4G), resembling the transcriptome of isolated human glomeruli (Fig. S5A). *NPHS2* gene expression was not affected by the *NPHS2* mutations. Both *NPHS2* mutations result in protein retention in the endoplasmic reticulum and do not interfere with mRNA expression (Nishibori et al., 2004; Tory et al., 2014).

*In vivo*, podocytes are the major VEGF producers in the glomerular compartment, which acts via autocrine and paracrine signaling to maintain the glomerular filtration barrier (Chen et al., 2004). Autocrine VEGFA signaling regulates slit diaphragm proteins, including podocin (Guan et al., 2006). In line with this, we showed, using PROGENy, that repaired organoid podocytes displayed enriched VEGF pathway activity compared with mutant organoid podocytes (Fig. 4H, Fig. S5B). Repaired organoid podocytes closely resembled fetal podocytes with respect to VEGF pathway activity, whereas mutant organoid podocytes differed from fetal podocytes (Fig. S5B). We identified that fibroblast growth factor (FGF) receptor 4 (FGFR4) signaling is enhanced in repaired organoid podocytes compared with mutant organoid podocytes (Figs S5C and S6; adjusted *P*<0.05; Liberzon et al., 2015). FGF signaling is likely to be involved in podocyte differentiation and plays a role in podocyte recovery following injury (Reiser et al., 2014). All up- and downregulated genes in mutant versus repaired organoids are shown in Fig. S5C,D.

The transcription factor activity of repaired organoid podocytes showed a distinct signature compared with mutant organoid podocytes (Fig. 4I). The top 20 enriched TFs included, among others, PR domain zinc finger protein 5 (PRDM5), E74-like ETS transcription factor 4 (ELF4) and zinc finger protein 69 (ZNF69), which are involved in the regulation of genes involved in podocyte physiology (Sharmin et al., 2016; Wu et al., 2018). Altogether, we have successfully modeled congenital NS in organoids. The repaired *NPHS2* mutation resulted in restored podocin expression and nephrin localization, as well as the rescue of pathway and TF activities essential in podocyte physiology.

### Idiopathic NS modeling using kidney organoids shows downstream slit diaphragm signaling events and granule formation in podocytes

The protamine sulfate model is a known *in vivo* model of specific podocyte injury induction (Verma et al., 2016). Protamine sulfate abolishes the negative charge on podocytes, thereby inducing foot process effacement accompanied by changes in downstream slit diaphragm signaling, cytoskeleton rearrangements and activation of injury-associated mechanisms. The protamine sulfate model is reversible by heparin treatment, which ultimately restores podocyte charge, the cytoskeleton and normal foot process formation (Verma et al., 2016).

**Fig. 5.**
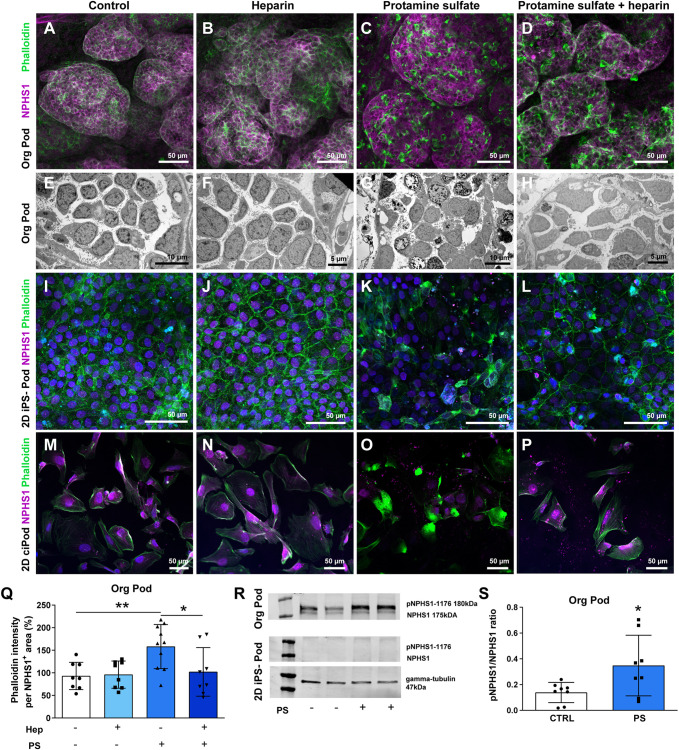
**Kidney organoids are the preferred model of choice to model idiopathic nephrotic syndrome.** (A-H) Podocyte cytoskeleton [phalloidin (green), NPHS1 (magenta)] (A-D) and ultrastructure analysis (E-H) of protamine sulfate-treated and heparin-rescued kidney organoid podocytes. (I-L) Podocyte cytoskeleton analysis [phalloidin (green), NPHS1 (magenta)] of protamine sulfate-treated and heparin-rescued 2D iPSC-derived podocytes. (M-P) Podocyte cytoskeleton analysis [phalloidin (green) and NPHS1 (magenta)] of protamine sulfate-treated and heparin-rescued 2D ciPODs. (Q) Quantification of phalloidin intensity in protamine sulfate-treated and heparin-rescued kidney organoid podocytes. *P<0.05, **P<0.01 (one-way ANOVA analysis followed by Tukey post-test). Data are presented as mean±s.d. from three independent experiments using at least two biological replicates per condition. (R) Western blotting analysis of pNPHS1 and NPHS1 protein expression in protamine sulfate (PS)-treated kidney organoids and 2D iPSC-derived podocytes. Gamma tubulin was used as a reference protein. (S) Semi-quantification of the pNPHS1/NPHS1 ratio of protamine sulfate-treated kidney organoids. *P<0.05 (unpaired *t*-test). Data are presented as mean±s.d. from three independent experiments using at least two biological replicates per condition.

Organoids treated with protamine sulfate showed clear podocyte cytoskeleton rearrangements (Fig. 5C,Q) and podocyte injury, as shown by vacuole formation and cell degeneration at the ultrastructural level (Fig. 5G) compared with controls (Fig. 5A,B,E,F). The protamine sulfate-induced injury could largely be reversed by heparin treatment (Fig. 5D,H,Q). iPSCs differentiated into 2D podocytes, from the same iPSC line as used for organoids, as well as ciPODs also showed protamine sulfate-induced cytoskeleton rearrangements (Fig. 5K, Fig. S7), which also showed partial recovery after heparin treatment (Fig. 5L, Fig. S7). Interestingly, only the organoids showed nephrin and protamine sulfate-induced phospho-nephrin expression upon western blotting (Fig. 5R,S), which is indicative of slit diaphragm signaling following injury, whereas nephrin expression could not be detected in 2D iPSC-derived podocytes or in ciPODs (Fig. 5R, Fig. S7).

To investigate further the preferred NS model of choice, i.e. 2D iPSC-derived podocytes versus 3D iPSC-derived organoids, the podocin mutant and repaired iPSC lines were evaluated in both models. *NPHS2* mutant and repaired organoids were exposed to protamine sulfate and/or treated with heparin. In line with our previous results, these organoids showed cytoskeleton rearrangements as a result of protamine sulfate exposure (Fig. S8A,F), which was only significantly reversed upon heparin treatment in podocin-repaired organoids (Fig. S8F).

Moreover, pNPHS1 expression in both organoid lines was not significantly induced upon protamine sulfate treatment. Nevertheless, a slight trend in pNPHS1 expression in the repaired line could be observed (Fig. S8B,C,G,H). Both mutant and repaired iPSCs, differentiated in 2D culture towards podocytes, responded to protamine sulfate resulting in injury, which recovered upon heparin treatment as shown by actin cytoskeleton expression (Fig. S8E,J). However, no nephrin and pNPHS1 expression was observed in 2D iPSC-derived podocytes (Fig. S8D,I), which agrees with our earlier results. These results support the suggestion that 3D organoids are the preferred model of choice to study podocytes accurately *in vitro*.

Using protamine sulfate, the early events affecting podocyte injury through cytoskeleton remodeling and affected slit diaphragm signaling can be modeled *in vivo* (Verma et al., 2016) and in kidney organoids (this study). The protein sulfate model is, however, an acute podocyte injury model. Therefore, we investigated the puromycin aminonucleoside (PAN) model and compared PAN-induced organoid podocyte injury to human minimal change disease NS pathology.

**Fig. 6.**
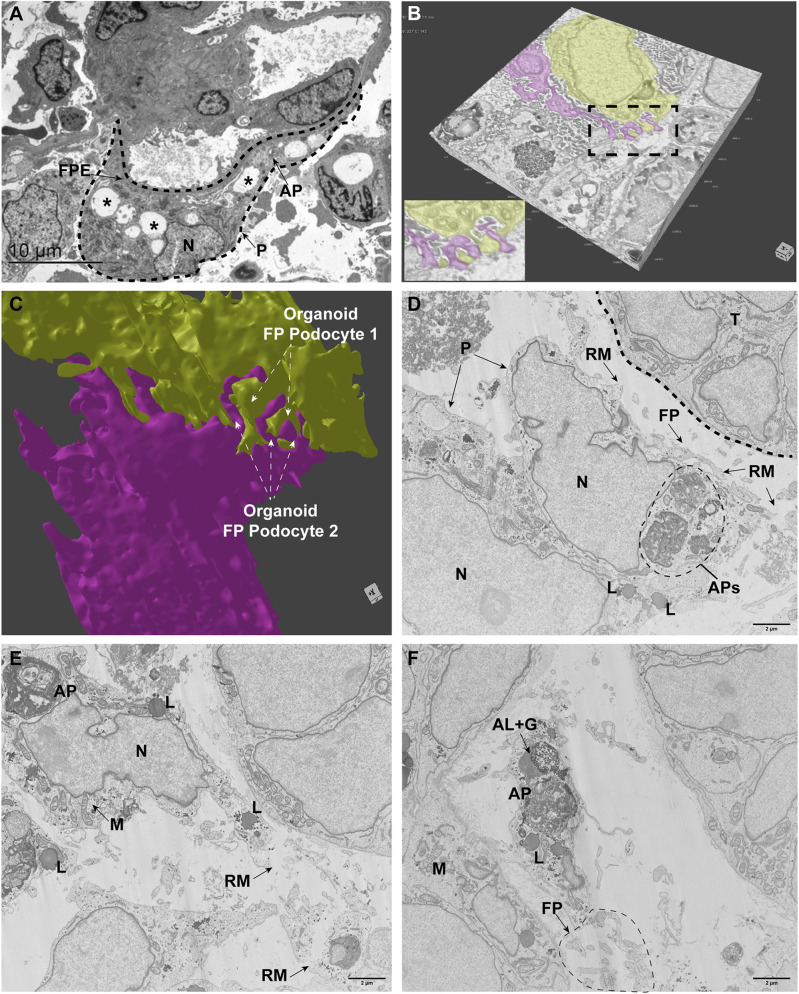
**Puromycin aminonucleoside-induced injury in kidney organoids results in autophagic activity comparable to human minimal change disease NS.** (A) Ultrastructural analysis of a human MCD kidney biopsy specimen as observed by TEM. The podocyte (dashed line, P) showed vacuole formation (examples marked with asterisks), foot process effacement (FPE) and autophagosomes (AP). N, nucleus. (B) 3D volume rendering of vehicle-treated kidney organoid, imaged by FIB-SEM, showing podocytes (yellow, magenta) and foot processes. The complete *z*-stack, including annotations, is shown in Movie 1. (C) 3D rendering of the segmented podocytes shows interdigitating podocyte foot processes (arrows). See Movie 2 for 3D rendering following segmentation. (D-F) FIB-SEM volume imaging (stack slide numbers: D, 327; E, 735; F, 1456) from PAN-injured podocytes (P) showing autophagosomes (APs), lysosomes (L), foot processes (FP) with ruptured membranes (RM). AL+G, autolysosome with glycogen; M, mitochondria; N, nucleus; T, tubular cells. Scale bars: 2 µm. The complete *z*-stack, including annotations, is shown in Movie 3.

Using focus ion beam scanning electron microscopy (FIB-SEM) volume imaging and transmission electron microscopy (TEM) we analyzed vehicle and PAN-treated (25 µg/ml, 24 h exposure) organoids and compared the results with kidney biopsy tissue from a patient suffering from minimal change disease (MCD). In human MCD, vacuoles, autophagosomes and foot process effacement were observed (Fig. 6A). FIB-SEM volume imaging of the vehicle-treated organoid podocytes showed polarized cell bodies, foot processes and filtration slits (Fig. 6B, Movie 1). Segmentation and 3D-rendering analysis confirmed interdigitating foot processes between podocytes (Fig. 6C, Movie 2). FIB-SEM volume imaging of the PAN-injured organoid podocytes showed autophagic activity in multiple podocytes, as indicated by autophagosomes and autolysosomes (Fig. 6D-F, Movie 3). Moreover, parts of the podocyte cell membrane were ruptured and podocyte foot processes were affected. These findings in organoids are in line with previous human studies showing that podocyte autophagy is correlated with podocyte foot process effacement and proteinuria in MCD NS (Ogawa-Akiyama et al., 2020; Zeng et al., 2014). These results demonstrate that organoid podocytes show similar pathological characteristics compared with podocyte damage in patients suffering from MCD, thereby emphasizing the potency of organoids for accurately modeling podocyte changes that lead to NS in patients.

Next, as a proof of concept, we showed that organoid podocytes respond to CPFs present in plasma from patients with NS recurrence after kidney transplantation (Fig. S9). We treated organoids for 4 h with patient plasma collected during plasmapheresis in the active course of the disease and plasma obtained after remission. Control plasmapheresis plasma was collected from an iNS patient after kidney transplantation, with no disease recurrence (plasma presumably free of CPFs). By flow cytometry analysis, we could show that NPHS1^+^ organoid podocytes exhibited increased cytoplasmic granule formation when exposed to active plasma compared with control and remission plasma (Fig. S9A). Podocyte granule formation is an acknowledged marker for podocyte injury (den Braanker et al., 2021; Seckin et al., 2017; Yamamoto-Nonaka et al., 2016). Podocyte morphology and nephrin protein expression were not affected after 4 h plasma exposure (Fig. S9B,C). In addition, we exposed organoids to plasma for 36 h and observed the initiation of cytoskeleton remodeling by actin condensation at the podocyte basement membrane (Fig. S9D). Our data suggest that organoid podocytes might be a useful model for investigating podocyte pathophysiology activated by CPFs and, ultimately, may contribute to the identification of CPFs involved in iNS.

## DISCUSSION

In the present study, we successfully modeled congenital (*NPHS2* mutant) and idiopathic NS using kidney organoids. Using iPSCs derived from a patient with two heterozygous podocin mutations, we could show the absence of podocin expression and aberrant nephrin localization in kidney organoids, which was rescued by CRISPR/Cas9 stem cell gene editing prior to differentiation into kidney organoids. As a result, VEGFA pathway and transcription factor activity, essential for podocyte physiology, were restored. iNS was modeled in organoids using plasma from a patient with post-transplant disease recurrence that resulted in podocyte injury. Moreover, we induced iNS-associated podocyte injury using protamine sulfate and PAN. We report that kidney organoids show superior morphology and expression of podocyte markers and activate downstream signaling cascades upon injury, which are absent in 2D iPSC-derived podocytes and human ciPODs, thereby making kidney organoids the preferred *in vitro* model to study podocyte injury that results in NS in patients.

NS pathophysiology is complex and the molecular mechanisms underlying the disease are still poorly understood, mainly owing to a lack of accurate human models. From previous studies, it is known that the podocyte is the main culprit in NS, consequently affecting glomerular filtration barrier integrity and leading to endothelial cell injury (Hansen et al., 2020; Rinschen et al., 2018; Smeets et al., 2011; Wharram et al., 2005). For decades, murine and human ciPODs have been used to unravel NS pathophysiology. Although these models have expanded our knowledge regarding podocytopathies in NS, we and others showed that *in vitro* 2D conditionally immortalized podocyte models do not fully exhibit *in vivo* podocyte characteristics (Hagmann and Brinkkoetter, 2018; Hale et al., 2018; Rinschen et al., 2018; van den Broek et al., 2021; Yoshimura et al., 2019). Here, we showed that kidney organoids are a promising tool to study both congenital and idiopathic NS because organoid podocytes express essential proteins, have a morphology closely resembling *in vivo* podocytes, and respond similarly to injury triggers. The 3D organoids show a superior expression profile compared with the 2D iPSC-derived podocytes. In 2D culture, nephrin is absent or the localization is still intracellular rather than between the interdigitating foot processes. Also, downstream filtration slit diaphragm signaling is absent in injured 2D iPSC-derived podocytes, emphasizing the limited differentiation properties of podocytes in 2D culture. It is known that 3D glomerulus-like structures isolated from organoids maintain marker expression for 96 h, whereas 2D podocytes migrating from these organoid glomeruli showed a clear alteration in their transcriptome (Hale et al., 2018). There is an unmet need for better *in vitro* models to recapitulate the *in vivo* glomerular filtration barrier, with emphasis on the podocyte. The 3D configurations and the role of mechanical as well as extracellular matrix (ECM) cues that influence cellular (de)differentiation in the development of accurate organoid models need to be addressed in order to establish improved glomerular models *in vitro*. The effect of ECM cues and material stiffness on organoid differentiation was nicely shown by Garetta et al. and also by Geuens and co-workers (Garreta et al., 2019; Geuens et al., 2021). Organoids encapsulated in soft biomaterials showed accelerated differentiation, less fibrosis upon aging, and reduced off-target cell populations, all aspects that will aid the signaling required to enhance the maturation of podocytes, but also other kidney segments such as the proximal tubule and the collecting duct.

Kidney organoids hold great promise as the next step in NS modeling; nevertheless, tissue maturity and the presence of off-target cells are often criticized. To overcome maturation challenges, organoid transplantation *in vivo* or onto chicken chorioallantoic membranes can be performed (Garreta et al., 2019; Sharmin et al., 2016; van den Berg et al., 2020, 2018). Organoid maturation can be achieved by the use of renal subcapsular transplantation of human kidney organoids in immunodeficient mice for the adaptive immune system, which results in size-selective filtration across the organoid glomerular filtration barrier (van den Berg et al., 2020). This transplantation approach emphasizes the potential of organoids to develop a mature glomerulus, which would be useful for studying a wide range of glomerular disorders; however, animals would still be required, which hampers high-throughput screens and is in conflict with the current attention for animal welfare. Altogether, when focusing on podocytopathies such as NS, the organoids we present in this study represent a useful tool to model idiopathic and congenital injury responses.

Despite the fact that podocytes are one of the best differentiated cell types in kidney organoids, the human adult podocyte signature is not yet fully accomplished in kidney organoids (Hale et al., 2018; Tran et al., 2019; Yoshimura et al., 2019). Our computational analysis revealed heterogeneity throughout the organoid podocyte populations based on collagen IV alpha chain expression. It is known that at the comma, S-shaped, and early capillary loop stages of glomerulogenesis, podocyte progenitors express collagen IV alpha chains 1 and 2. This collagen IV network is replaced by collagen IV alpha chains 3, 4 and 5 at the beginning of the capillary loop stages and, ultimately, these chains are solely expressed by mature podocytes (Abrahamson et al., 2009). The exact mechanism of the collagen IV isoform switch remains elusive, although it has been described to aid in podocyte differentiation and is likely under the control of VEGF, which in turn is regulated by, among others, PI3K signaling (Chen et al., 2004; Sison et al., 2010; Veron et al., 2012). In line with this, we showed that podocyte subcluster 3 had increased collagen IV alpha 3 and VEGF expression as well as PI3K signaling compared with the other two podocyte subclusters. Moreover, solely cluster 3 showed ligand-receptor interactions with endothelial cell precursor receptors, as a potential first step towards glomerular filtration barrier formation, thereby confirming that cluster 3 is more mature compared with the other two podocyte clusters. Cluster 3 resembled in part adult podocytes in terms of collagen IV alpha 3 expression. The data we collected in this study could aid in establishing the culture conditions required to achieve a fully mature podocyte population in organoids.

The PI3K signaling pathway, which regulates VEGF signaling, can be induced by tyrosine kinases such as epidermal growth factor receptor (EGFR) (Pages and Pouyssegur, 2005), which is achieved in our kidney organoid culture protocol by EGF supplementation. Despite the EGFR stimulation, the consequent PI3K activation did not result in homogenous VEGF expression levels as VEGF expression varied between the podocyte clusters we identified in our organoids. Also, the TF signature in podocyte cluster 3 showed closest, but still incomplete, resemblance with adult podocytes. To accelerate the differentiation of podocytes, VEGF signaling is likely one of the major actors (Eremina et al., 2003).

To stimulate VEGF signaling, dynamic modulation of WNT signaling has been shown to be a promising approach to enhance podocyte differentiation in kidney organoids and corresponding VEGFA levels. Consequently, enhanced VEGFA levels promoted a defined vascular network, which ultimately is essential for glomerular filtration barrier maturation (Low et al., 2019). When focusing on VEGF signaling regulation, a magnitude of factors are involved. The promoter region of VEGF contains multiple trans-activating factors and responsive elements (Pages and Pouyssegur, 2005), such as hypoxia inducible factor-1 (HIF1), that might open avenues for transcriptional stimulation of VEGF expression throughout all podocytes present in the organoids. Notably, organoids are cultured under normoxic conditions, in contrast to glomerulogenesis *in vivo*, which occurs under hypoxic conditions (Ryan et al., 2021). A hypoxic environment will activate HIF transcription factors, regulate VEGF signaling, and consequently stimulate podocyte differentiation (Ryan et al., 2021). Mimicking the delicate balance of VEGF signaling during the different stages of nephrogenesis will be a challenge *in vitro* (Eremina et al., 2003). Obviously, podocyte maturation not only depends on VEGF signaling but relies on a plethora of factors. For instance, podocyte maturation also relies on the concerted actions with other cell types, such as podocyte-macrophage crosstalk (Munro et al., 2019), fluid flow, including nutrient exchange, as well as microenvironmental ECM cues (Garreta et al., 2019; Geuens et al., 2021; Homan et al., 2019; Ryan et al., 2021; van den Berg et al., 2020).

In conclusion, we report successful patient-based idiopathic and congenital podocytopathy modeling in iPSC-derived human kidney organoids. We showed that 3D kidney organoids are the model of choice for investigating podocyte changes that result in NS in patients, as confirmed in three iPSC lines, compared with 2D cultured podocytes, and in particular that a specific podocyte cluster in part resembles adult podocytes. Kidney organoids represent a powerful tool for studying underlying NS disease mechanisms, may have the potential to identify pathogenic CPFs in patients' plasma, and allow for the study of functional genomics. Altogether, kidney organoids will help to improve our understanding of podocytopathies and may be used for developing novel effective therapies to treat NS. Once a mature glomerular filtration barrier is developed *in vitro*, this high-end platform could be used beyond studying podocytopathies and may have the potential to become an accurate model for resolving the pathophysiology of a wide range of glomerular disorders.

## MATERIALS AND METHODS

### Ethical statement

Human adult skin fibroblasts derived from a healthy volunteer and erythroblasts from a pediatric patient suffering from congenital NS, after giving informed consent, were used to generate iPSCs. This study was conducted in accordance with the Helsinki Declaration as revised in 2013. Permission for the creation and use of iPSCs in this study was obtained from the local ethical commission for human-related research of the Radboud University Medical Center, Nijmegen (approval numbers: 2015-1543 and 2006-048). Human kidney tissue was used for immunostaining and EM analysis. Ethical permission for the use of archived human kidney material was given by the local commission for human-related research of the Radboud University Medical Center, The Netherlands (approval number: 2018-4086).

### Patient and healthy control plasma

This study was approved by the regional medical-ethical committee (Arnhem-Nijmegen), under file number 09/073, and has been carried out in compliance with the Declaration of Helsinki as revised in 2013. All human participants signed informed consent prior to the study, as specified in the International Committee of Medical Journal Editors recommendations.

### Reagents and resources

Reagents and resources (e.g. antibodies, chemicals, commercial assays, software and algorithms) are listed in Table S5.

### Cell culture

#### iPSC generation

Healthy control iPSCs were generated using fibroblasts from a healthy volunteer at the Radboudumc Stem Cell Technology Center through the use of lentiviral vectors, previously described by Warlich et al. (2011). In short, *Escherichia coli* were used to clone lentiviral vectors containing the transcription factors Oct4, Klf4, Sox2 and cMyc together with the fluorescent marker dTomato (pRRL.PPT.SF.hOKSMco-idTom.pre.FRT). HEK293T cells were then used for viral production using packaging (pCMV-VSV-G) and envelope (pCMV-dR8.91.) vectors, respectively. Fibroblasts were transduced with viral supernatants and re-seeded on plates with inactivated mouse embryonic fibroblast (MEF) feeder cells to support the growth and pluripotency of iPSCs. Emerging iPSC colonies were picked, expanded and assessed for activation of stem cell markers to confirm pluripotency.

Mutant iPSCs, containing two mutations in the podocin-encoding gene {*NPHS2*; Chr1(GRCh37); NM_014625.3:c.413G>A [p.(Arg138Gln); heterozygote] and c.478G>T [p.(Asp160Tyr); heterozygote]} were generated from erythroblasts from a pediatric patient using the CytoTune™-iPS 2.0 Sendai Reprogramming Kit (Thermo Fisher) according to the manufacturer's protocol. In short, erythroblasts were transduced using the CytoTune™-iPS 2.0 Sendai Reprogramming Kit and re-seeded on plates with inactivated MEFs to support the growth and pluripotency of iPSCs. Emerging iPSC colonies were picked, expanded and assessed for activation of stem cell markers to confirm pluripotency.

#### iPSC maintenance culture

iPSCs were cultured using Essential™ 8 (E8) medium (Gibco, Thermo Fisher) supplemented with E8 supplement (50×, Gibco) and 0.5% (v/v) antibiotic-antimycotic (Gibco) on Geltrex-coated (Thermo Fisher) cell culture plates (Greiner). Upon 70-90% confluency, iPSCs were washed three times with PBS and subsequently passaged in colonies using 0.5 mM EDTA (Thermo Fisher) in PBS for 5 min at room temperature (RT). For cell seeding, iPSCs were washed three times with PBS and subsequently disassociated into single cells using TrypLE Select Enzyme (Thermo Fisher) for 2 min at 37°C.

#### ciPOD culture

The ciPODs used in this study was kindly provided by Prof. Saleem (University of Bristol, UK) (Saleem et al., 2002). ciPODs were cultured using uncoated 75 cm^2^ culture flasks (Greiner) at 33°C with 5% (v/v) CO_2_ in DMEM/F12 (Gibco) supplemented with 5 µg/ml insulin, 5 µg/ml transferrin, 5 ng/ml selenium (Sigma-Aldrich), 10% heat-inactivated fetal calf serum (Gibco) and 1% (v/v) penicillin/streptomycin (Gibco). Passaging and seeding of cells was performed by washing cells with PBS and subsequently detaching them using Accutase^®^ (Sigma-Aldrich), according to the manufacturer's protocol. ciPODs were seeded at 62,500/cm^2^ in black flat-bottom 96-well plates (Corning) for immunofluorescence imaging or 6-well plates (Greiner) for protein and RNA isolation and were incubated at 37°C with 5% (v/v) CO_2_, which initiated differentiation of the ciPODs.

All cell lines used tested negative for contamination, including *Mycoplasma*.

### Differentiation protocols

#### 2D podocyte differentiation

An adapted protocol based on Rauch et al. and Musah et al. (Musah et al., 2017; Rauch et al., 2018) was used to differentiate iPSCs into podocytes. iPSCs were seeded at 15,000 cells/cm^2^ on Geltrex-coated cell culture plates in E8 medium supplemented with E8 supplement, 0.5% (v/v) antibiotic-antimycotic and 1× RevitaCell (Gibco). One day later, defined as day 1, differentiation of iPSCs into metanephric mesenchyme was started by adding Essential™ 6 (E6) medium (Gibco), supplemented with 15 ng/ml human recombinant bone morphogenetic protein 7 (R&D Systems), 10 ng/ml human recombinant activin A (R&D Systems), 100 nM retinoic acid (Sigma-Aldrich) and 1× non-essential amino acids (Gibco). At day 11, podocyte differentiation was started by culturing cells in E6 medium supplemented with 1× non-essential amino acids and 10 ng/ml vascular endothelial growth factor subtype A (R&D Systems). At day 20, iPSC-derived podocytes were mature and used for experiments.

#### 3D organoid differentiation

iPSCs were seeded on Geltrex-coated cell culture plates using E8 medium supplemented with E8 supplement, 0.5% (v/v) antibiotic-antimycotic and 1× RevitaCell at a density of 18,750-22,900 cells/cm^2^. Twenty-four hours after seeding, culture medium was replaced by E6 medium supplemented with 6 µM CHIR 99021 (Tocris, Bio-Techne) to initiate differentiation (defined as day 0). From day 3 onwards, half of the wells were cultured in E6 medium supplemented with 200 ng/ml FGF9 (R&D Systems) and 1 µg/ml heparin (Sigma-Aldrich) until day 7 (anterior intermediate mesoderm; AM), whereas the other wells remained on E6 medium with CHIR 99021 until day 5 (posterior intermediate mesoderm; PM). From day 5 onwards, all cells were cultured in E6 medium with FGF9 and heparin. At day 7, cells were harvested using trypsin-EDTA and a cell suspension was made consisting of AM and PM cells in a 1:2 ratio. Cells aggregates, consisting of 300,000 cells, were transferred to Transwell™ plates (Corning) and incubated for at least 1 h at 37°C, 5% (v/v) CO_2_, with E6 medium supplemented with 5 µM CHIR 99021 to induce self-organizing nephrogenesis. After 1 h, medium was replaced with E6 medium supplemented with FGF9 and heparin. At day 7+5 (i.e. 5 days later), medium was changed to E6 supplemented with EGF (10 ng/ml, R&D Systems) and BMP7 (50 ng/ml, R&D Systems). Organoids were used for experiments at day 7+17 or 7+18.

### Protamine sulfate exposure and heparin rescue

Organoids, ciPODs and 2D iPSC-derived podocytes were exposed to protamine sulfate treatment and rescued with heparin. Briefly, protamine sulfate (2 mg/ml, Sigma-Aldrich) was dissolved in E6 medium and organoids and 2D cell cultures were exposed at 37°C, 5% (v/v) CO_2_ for 2 h. After 2 h, cells (organoids and 2D cells) exposed to protamine sulfate were processed for protein isolation and immunofluorescence staining, as described in the following sections. Sequentially, protamine sulfate-injured organoids and 2D cell cultures were exposed to heparin (800 µg/ml in E6, heparin sodium salt from porcine intestinal mucosa; Sigma-Aldrich) at 37°C, 5% (v/v) CO_2_ for 2 h. Finally, after 2 h, these organoids and cell cultures (groups: control, heparin control, protamine sulfate plus heparin treatment) were also processed for protein isolation and immunofluorescence staining.

### Puromycin aminonucleoside treatment of organoids and sample preparation for electron microscopy and FIB-SEM volume imaging

Kidney organoids were exposed to vehicle or puromycin aminonucleoside (25 µg/ml, MedChemExpress) at 37°C, 5% (v/v) CO_2_ for 24 h. Next, organoids were washed in PBS and fixed using 2.5% (w/v) paraformaldehyde (PFA) (Sigma-Aldrich) +0.1% (v/v) glutaraldehyde [Electron Microscopy Sciences (EMS)] in 1 mM phosphate buffer, at pH 7.4. After 1 h the samples were transferred to 4% (v/v) PFA in 1 mM phosphate buffer, pH 7.4 for 48 h. After fixation, the samples were stored in 0.1 M cacodylate buffer (EMS) at 4°C until further processing for electron microscopy. To enhance the sample contrast for electron microscopy, an OTO-based protocol was used (Seligman et al., 1966; Tapia et al., 2012). Briefly, the samples were incubated in 2% (w/vl) osmium tetroxide [Structure Probe, Inc. (SPI)]/1.5% (w/v) potassium ferrocyanide (Merck) in 0.1 M cacodylate buffer with 2 mM CaCl_2_ (Merck) for 1 h at RT, and then washed and incubated in 0.5% (w/v) thiocarbohydrazide solution (Sigma-Aldrich) for 30 min at RT. After rinsing, the samples were incubated again in 2% (w/v) osmium tetroxide for 30 min at RT, and then rinsed and incubated in 2% (w/v) aqueous uranyl acetate (EMS) at 4°C overnight. After washing, the samples were incubated in lead aspartate (Merck) solution (pH 5.5) for 30 min at 60°C, rinsed and dehydrated in ascending ethanol series. The dehydrated samples were then transferred into a mixture of acetone and Durcupan (Sigma-Aldrich) at 3:1 (for 1.5 h), 1:1 (for 1.5 h), then 1:3 overnight. Subsequently, the samples were incubated in 100% Durcupan for 4 h before embedding and polymerization with fresh Durcupan.

After polymerization, semi-thin sections (500 nm) were cut with a Reichert Ultracut S ultramicrotome (Leica) and stained with Toluidine Blue (Sigma-Aldrich). The region of interest was cut out of the resin block and glued on an SEM stub with carbon tape, using conductive silver paint. The samples were coated with a gold sputter coater (Edwards, Stockholm, Sweden) before introduction into a Zeiss Crossbeam 550 FIB-SEM (Carl Zeiss).

Multiple coarse trenches were milled (using a 30 kV@30 nA probe) to choose the regions of interest for further 3D volume imaging. Parameters for serial sectioning imaging of large regions were set using the Atlas 3D software (Atlas Engine v5.3.3). The large trenches were first smoothened using a 30 kV@1.5 nA FIB probe, and thereafter a 30 kV@700 pA probe current was used for serial FIB milling. InLens secondary and backscattered electron microscopy images were simultaneously collected at an acceleration voltage of 2.0 kV with a 300 pA probe current. The backscattered grid was set to −876 V. For noise reduction, images were acquired using line average (*n*=4) and a dwell time of 0.7 ms. The milling and imaging processes were continuously repeated and long series of images were acquired, creating original stacks of 30×24×12 µm for both the control and PAN-injured samples. The imaging voxel size in both cases was set to 5×5×10 nm.

#### Image post processing and segmentation

The FIB-SEM images were processed using MATLAB (MathWorks) and ImageJ (National Institutes of Health) to correct for uneven illumination, misalignment, de-striping and contrast enhancement. A summary of each processing step is given below.

##### Alignment

Consecutive slices were aligned using normalized cross correlation. Briefly, the first image in the stack was chosen as reference and the second image was translated pixel by pixel across the reference and a normalized cross-correlation matrix was obtained using the ‘normxcorr2’ function. The location of the highest peak in the cross-correlation matrix (representing the best correlation) was then used to calculate the translation required to align the two images. Once the moving image was aligned with the reference image, it served as the reference for alignment of the subsequent slice.

##### Uneven illumination

In order to obtain a homogeneous background, the uneven illumination between the upper and lower half of the images was corrected using the ‘rolling ball background subtraction’ feature in ImageJ. No smoothing was performed during subtraction.

##### Histogram matching

In order to avoid losing the color depth and the original contrast and brightness of the images after background correction, the histogram of each image was matched with its corresponding original image using the ‘imhistmatch’ function in MATLAB with ‘polynomial’ mapping method. The number of bins chosen was equal to the number of bins in the original image.

##### Vertical stripes

Removal of the vertical stripes in the stacks was performed following a wavelet-FFT filtering approach described by Münch et al. (2009). Briefly, the high-frequency information corresponding to the vertical stripes was successively condensed into a single coefficient map using decomposition by ‘coif’ wavelet family. Subsequently, a 2D-fourier transformation was performed to further tighten the stripe information into narrow bands. Finally, the condensed stripe information was eliminated by multiplication with a gaussian damping function and the de-striped image was reconstructed by inverse wavelet transform.

##### 3D segmentation

Dragonfly™ image analysis and deep-learning software (Objects Research Systems, version 2020.1) was used to segment all image data. For efficient interpolation during segmentation, the images were first binned by a factor of two in the *x* and *y* directions (image plane) resulting in a pixel size of 10×10 nm. Subsequently, the stack was down-sampled by a factor of two in all directions, resulting in a cubic voxel size of 20×20×20 nm.

### TEM

Organoids were fixed overnight using 2.5% (w/v) glutaraldehyde dissolved in 0.1 M sodium cacodylate. Tissue fragments were then fixed for 1 h using 2% OsO_4_ buffered in Palade's buffer [0.23 % (w/v) CH_3_COONa, 0.58% (w/v) C8H11N_2_NaO_3_ dissolved in MilliQ (pH 7.3)]. Tissue was dehydrated through an increasing series of alcohol and embedded in Epon 812 (Sigma-Aldrich). Using a Leica Ultracut, 90 nm ultra-thin sections were cut, mounted on copper grids and contrasted at RT with uranyl acetate and lead citrate. Sections were assessed using a Jeol JEM 1400 electron microscope at 60 kV. For image collection, a digital camera was used (Gatan).

### Preparing paraffin sections of human iPSC-derived kidney organoids

iPSC-derived kidney organoids were cut from the Transwell™ filter and fixated in 4% (v/v) formalin on ice for 20 min. Fixed iPSC-derived kidney organoids were stripped of the filter membrane using a scalpel. Multiple organoids from similar conditions were stacked on top of each other and embedded in a cryomold (Tissue-Tek, Sakura Finetek Europe B.V.) using 2.25% (w/v) agarose gel (Thermo Fisher). After embedding for 5 min at 4°C, the iPSC-derived kidney organoids were transferred to embedding cassettes and paraffinized. After paraffinization, iPSC-derived kidney organoids were cut at a thickness of 4 µm using a rotary microtome (Microm HM355 S, GMI) and mounted on FLEX IHC microscope slides (DAKO, Agilent Technologies).

### Immunofluorescence staining

#### Paraffin tissue

Using a series of xylol (2×) and 100% (v/v) ethanol (3×), paraffin slides were deparaffinized. Antigen retrieval was performed by boiling slides in Tris-buffered EDTA (VWR Chemicals) for 10 min at 180 W in a microwave. Primary (1:100) and secondary (1:200) antibodies were diluted in PBS containing 1% (v/v) bovine serum albumin (BSA; Sigma-Aldrich). Primary antibodies were incubated overnight at 4°C, and secondary antibodies were incubated at RT for 2 h. After each antibody incubation, slides were washed three times in PBS for 5 min. Slides were mounted in Fluoromount-G^®^ (Southern Biotech, SanBio). Primary and corresponding secondary antibodies are listed in Table S2. Images were captured using a Zeiss LSM 880 confocal microscope or Leica DMI6000B high-content microscope.

#### Whole-organoid staining

Whole-mount staining was performed according to Takasato et al. (2016). Briefly, organoids were fixed using 2% (w/v) PFA at 4°C for 20 min. After a PBS wash, organoids were blocked in blocking buffer containing 10% (v/v) donkey serum (GeneTex) and 0.6% (v/v) Triton X-100 in PBS at RT for 2 h. Primary antibodies (Table S2) were diluted 1:300 and incubated at 4°C overnight. Next, organoids were washed in PBTX [0.3% (v/v) Triton X-100 in PBS]. Secondary antibodies (Table S2) were diluted 1:400 in PBTX and incubated at RT for 2 h. Organoids were washed using PBS and mounted using Fluoromount-G^®^. Images were captured using a Zeiss LSM 880 confocal microscope.

### Organoid filtration slit super-resolution imaging

Sample processing and subsequent imaging were performed as described previously (Artelt et al., 2018). In brief, 4 µm paraffin sections were directly mounted on coverslips (VWR). To correct for PFA-induced autofluorescence, samples were incubated with 100 mM glycine in PBS for 10 min. Samples were blocked with 1% (v/v) fetal bovine serum (FBS), 1% (v/v) goat serum, 1% (v/v) bovine albumin and 0.1% (v/v) cold fish gelatin in PBS at RT for 1 h. Primary antibody against nephrin (guinea pig, PROGEN, GP-N2, 1:100) was diluted in blocking solution and detected by a secondary anti-guinea pig Cy3-antibody (Jackson ImmunoResearch Laboratories, 1:800) that was also diluted in blocking solution. 3D-SIM images were acquired using a Zeiss Elyra PS.1 system. Using Zeiss ZEN black software, 3D-SIM images were reconstructed. As control *in vivo* tissue, we used sections of male Munich Wistar Frömter rats, age ranging from 41-54 weeks. The animals were housed under standard conditions. Animal procedures were approved by German government officials (LANUV NRW 50.203.2 – AC 10/06) and performed in accordance with the European Communities Council Directive (86/609/EEC).

#### 2D podocyte staining

iPSC-derived podocytes and ciPODs cultured in 96-well plates were fixed in 2% (w/v) PFA for 10 min at RT. Cells were blocked in blocking buffer (same as used for whole organoid stain) at RT for 2 h. Primary antibodies (Table S2) were diluted 1:100 and incubated at 4°C overnight. Next, cells were washed using PBTX and then incubated with secondary antibody diluted 1:200 in PBTX at RT for 2 h. Cells were washed and mounted using Fluoromount-G^®^. Images were captured using a Leica DMI6000B high-content microscope.

### Protein harvesting, gel electrophoresis and western blot

Tissue was collected and incubated on ice for 60 min using RIPA buffer containing 1× RIPA containing a phosphatase inhibitor Na_3_VO_4_ (Cell Signaling Technology), 1 mM phenylmethylsulfonyl fluoride (PMFS, Sigma-Aldrich) and 2% (w/v) sodium dodecyl sulphate (SDS; Sigma-Aldrich) in PBS. After incubation, tissue was centrifuged at 16,000 ***g*** for 15 min at 4°C. The supernatant containing protein was collected and stored at −80°C for further processing. Protein samples were mixed 1:4 with 4× Laemmli buffer (Bio-Rad) containing 10% (v/v) β-mercaptoethanol (Merck) and incubated at 95°C for 5 min. Samples were spun down and loaded onto a 4-20% (w/v) polyacrylamide precast mini-PROTEAN TGX gel (Bio-Rad) for subsequent gel electrophoresis. Gels were then transferred onto a nitrocellulose blotting membrane (Amersham, GE Healthcare) and blocked for 1 h at RT using Odyssey blocking buffer (LI-COR Biosciences, Bio-Rad) in a 1:1 dilution with Tris-buffered saline (TBS) containing 0.1% (v/v) Tween^®^ 20 (Merck) (TBS-T). Subsequently, membranes were washed three times for 5 min each with TBS-T and incubated overnight with primary antibodies at 4°C. After washing the membranes again three times for 5 min each with TBS-T, membranes were incubated with corresponding secondary antibodies for 1 h at RT. Membranes were washed using TBS and subsequently imaged and semi-quantified using a LI-COR Odyssey CLx imaging system. Primary and corresponding secondary antibodies are listed in Table S3.

### Fluorescence-activated cell sorting (FACS)

Organoids cultured on Transwell™ plates were washed twice in PBS, dissociated using Accutase^®^ and transferred to sterile 6-well plates (without Transwell™ inserts). Organoids from similar culture conditions were pooled and incubated for 15 min at 37°C. During incubation, the cell suspension was resuspended every 2 min for homogenization purposes. After incubation, the cell suspension was put on ice for 1 min to inactivate the Accutase^®^, cells were strained using a 40 µm cell strainer (Corning) and the cell strainer was washed repeatedly with 10% (v/v) fetal calf serum in DMEM/F12 to collect all cells. The strained cell suspension was centrifuged at 250 ***g*** for 5 min at 4°C and the supernatant was discarded. Cells were washed with FACS buffer [1% (v/v) BSA in PBS] once and incubated with primary antibodies for NPHS1 (1:100, R&D Systems, AF4269) in FACS buffer for 45 min at 4°C. After incubation, cells were centrifuged at 250 ***g*** for 5 min at 4°C and again washed with FACS buffer. After washing, cells were again centrifuged at 250 ***g*** for 5 min at 4°C and subsequently incubated with corresponding secondary antibodies (1:200, donkey anti-sheep Alexa Fluor™ 647, Thermo Fisher, A-21448) for 30 min at 4°C. Cells were centrifuged at 250 ***g*** for 5 min at 4°C and washed twice in FACS buffer. After washing, cells were incubated with DAPI (300 nM, Thermo Fisher) in FACS buffer 10 min prior to FACS (for live/dead gating). Detection events were gated to select cells and doublet control was performed for both forward and side scatter. Living cells (DAPI^−^) were selected and gated on NPHS1^+^ (podocytes). Cells were sorted by immunofluorescence using a fluorescence cell sorter (BD Biosciences; FACSAria™ II SORP 18-color) and DIVA8 software. After cell sorting, DAPI^−^/NPHS1^+^ cells were collected in RLT buffer (RNeasy, Qiagen) for further processing.

### Plasma exposure and granule formation analysis using flow cytometry

D7+17 organoids were exposed to 10% (v/v) plasmapheresis plasma supplemented with anticoagulants heparin (100 µg/ml, Sigma-Aldrich) and PPACK (10 µM, Santa Cruz Biotechnology) and incubated at 37°C, 5% (v/v) CO_2_, for 4 h and 24 h. The plasma was collected during plasmapheresis of a patient with post-transplant recurrent NS, during the active course of the disease and after remission. After collection, plasmapheresis plasma was aliquoted and stored at −80°C. As controls, we used E6 culture medium, E6 medium supplemented with the anti-coagulants heparin (100 µg/ml) and PPACK (10 µM), and plasmapheresis plasma collected from an iNS patient after kidney transplantation, with no disease recurrence (plasma presumably free of CPFs) and supplemented with anticoagulants. Next, single organoids were washed with PBS and digested using Accutase^®^ for 15 min at 37°C; during this step, the suspension was resuspended every 5 min. Accutase^®^ was inactivated by adding DMEM/F12 supplemented with 10% (v/v) FBS. The organoid suspensions were centrifuged at 250 ***g*** for 5 min. Cells were washed once using wash buffer [PBS containing 1% (v/v) BSA] and again centrifuged. Next, the cells were incubated using a primary antibody against NPHS1 (R&D Systems, AF4269), diluted 1:100 in FACS buffer [PBS supplemented with 1% (v/v) BSA] for 1 h at 4°C. After two washing steps, the cells were incubated using a secondary antibody donkey anti-sheep Alexa Fluor 647™ (Thermo Fisher, A21448) 1:200 in FACS buffer for 45 min at 4°C. Cells were centrifuged at 250 ***g*** for 5 min at 4°C and washed twice in FACS buffer. After washing, cells were incubated with DAPI (300 nM, Thermo Fisher) in FACS buffer 10 min prior to FACS (for live/dead gating). Detection events were gated to select cells and doublet control was performed for both forward and side scatter. Living cells (DAPI^−^) were selected and gated on NPHS1^+^ (podocytes). Cells were measured with a flow cytometer (NovoCyte 3000 flow cytometer, Agilent), as previously described by den Braanker et al. (2021). The granule formation in living NPHS1^+^ podocytes was quantified by sideward scatter.

### Transcriptome analysis

#### scRNA-seq

D7+17 organoids were washed with PBS and digested using Accutase^®^ at 37°C for 15 min. Accutase^®^ was inactivated by adding DMEM/F12 with 10% (v/v) FBS. The cell suspension was filtered through a 40 µm cell strainer and spun down at 1250 ***g*** for 5 min. To reduce background and obtain a single-cell suspension, this step was repeated once more. Cells were then resuspended in PBS containing 0.04% (v/v) BSA and counted using a Neubauer counting chamber (Carl Roth). Next, 1000 cells/µl per sample were loaded onto a 10x Chromium Next GEM Chip G according to the manufacturer's instructions and processed in a Chromium controller (10x Genomics). All single-cell sequencing libraries were generated using 10x Chromium Next GEM v3.1 kits (10x Genomics) and sequencing was performed on a Novaseq6000 system (Illumina), using an S2 v1.5 100 cycles flow cell (Illumina) with run settings 28-10-10-90 cycles. After sequencing, Cell Ranger (v.3.1.0) was used to align the reads to the human genome GRCh38, using default settings.

#### Standard scRNA-seq analysis workflow

The pre-processed reads were analyzed using the R package Seurat (version 4.0.3) (Hao et al., 2021). Data was loaded using the ‘Read10X’ function and transformed into a Seurat object. A Seurat object was created with features (genes) that appeared in at least three cells and cells that contained at least 200 different features as a first filtering step. Next, cells were filtered out containing <200 or >3000 features to filter out damaged cells and doublets. Also, cells in which mitochondrial genes contributed for >20% to the total gene expression were believed to be damaged or apoptotic and therefore discarded. After these quality control checks, data were log normalized and variable features were identified using the ‘FindVariableFeatures’ function with the vst selection method. Afterwards, the Seurat package was used to scale the data, and run a principal component analysis (PCA) dimensionality reduction. Data from the PCA was used to construct a shared nearest neighbor (SNN) graph taking into account 20 principal components (PCs), which was subsequently used to identify clusters using the ‘FindClusters’ function with the resolution set to 0.7. For visualization, the ‘RunUMAP’ function was used. Next, cluster markers were obtained by running the ‘FindAllMarkers’ function. Annotation of the clusters was performed manually using these cluster markers, and cluster markers described in the literature and the Humphreys Kidney Interactive Transcriptomics (KIT) website (http://humphreyslab.com/SingleCell/).

#### scRNA-seq in-depth podocyte cluster analysis

For in-depth podocyte analysis, the podocyte cluster was extracted from the total dataset and variable features were calculated again for this subset only. Next, the same workflow as described for the complete dataset after identifying variable features was performed. For comparative analysis of the organoid podocyte subclusters (this manuscript) of adult and fetal podocytes, publicly available datasets obtained from the GEO database (Barrett et al., 2013) were used (assession numbers GSE118184 and GSE112570, respectively. Seurat objects were created from these datasets, and dotplots for NPHS1, NPHS2, podocalyxin, synaptopodin and Wilms' tumor 1 were used to define the podocyte population within each dataset. This cluster was subsetted and merged with the subsetted podocytes from the organoids. Podocyte datasets were integrated using the ‘runHarmony’ function from the Harmony (version 0.1.0) package (Korsunsky et al., 2019). Subsequently, we compared the podocyte clusters for marker expression using Seurat, pathway activity using PROGENy (version 1.14.0) and transcription factor activity using DoRothEA (version 1.4.1) and visualized using the pheatmap (version 1.0.12) R package (Garcia-Alonso et al., 2019; Holland et al., 2020a,b; Schubert et al., 2018). Cluster markers of podocyte clusters were determined using the ‘FindAllMarkers’ function on a dataset with only these podocyte clusters (organoid, adult and fetal). These cluster markers were filtered to retain only significant markers (adjusted *P*<0.05) and were divided into upregulated (log fold change>0) or downregulated (log fold change<0). A list of significantly upregulated cluster markers was used to perform GO and KEGG enrichment analysis, using the clusterProfiler package (version 4.0.2) (Yu et al., 2012). MSigDB enrichment analysis was done using the ‘H’ or ‘C2’ category gene set from the msigdbr package (version 7.2.1) (Liberzon et al., 2015).

#### Ligand-receptor interaction analysis

In order to assess cellular interactions between cellular clusters within the organoid, we replaced the podocyte cluster that was obtained by unsupervised clustering with the three podocyte subclusters that were identified during in-depth podocyte cluster analysis. Furthermore, cells positive for GATA3 and negative for CDH1 were named mesangial cells and cells positive for PECAM1 or CD34 or ICAM1 or CDH5 or PLVAP were named endothelial cells. Ligand-receptor analysis was performed with the addition of podocyte clusters 1, 2 and 3, and mesangial cells and endothelial cells added to the original clusters identified by unsupervised clustering using Seurat. scRNA sequencing matrices were log-normalized and scaled and used as input for ligand receptor inference by CellphoneDB (version 2.0.5) (Efremova et al., 2020). Analysis was performed using the statistical_analysis mode of the CellphoneDB package. Using statistically significant interactions (*P*<0.05) in the CellphoneDB output, ranking and visualization of the data was performed by CrossTalkeR (version 1.0.0) (Nagai et al., 2021). The same clusters were used to obtain GO, KEGG and Hallmark H enrichment in a similar way as for the podocyte clusters (organoid, adult and fetal) described above.

#### Bulk RNA sequencing

Sequencing was performed at Single Cell Discoveries, a (single cell) sequencing provider located in The Netherlands, using an adapted version of the CEL-seq protocol. In short, total RNA was extracted from FACS-sorted podocyte cell pellets using the Ambion PureLink RNA mini kit (Thermo Fisher) according to the manufacturer's protocol and used for library preparation and sequencing. mRNA was processed as described previously, following an adapted version of the scRNA-seq protocol of CEL-Seq (Hashimshony et al., 2012; Simmini et al., 2014). Briefly, samples were barcoded with CEL-seq primers during a reverse transcription and pooled after second strand synthesis. The resulting cDNA was amplified with an overnight *in vitro* transcription reaction. From this amplified RNA, sequencing libraries were prepared with Illumina Truseq small RNA primers. Paired-end sequencing was performed on the Illumina Nextseq500 platform. Read 1 was used to identify the Illumina library index and CEL-Seq sample barcode. Read 2 was aligned to the hg19 human RefSeq transcriptome using BWA (Li and Durbin, 2010). Reads that mapped equally well to multiple locations were discarded. Mapping and generation of count tables was carried out using the MapAndGo script1. Samples were normalized using RPM normalization.

#### Bulk RNA-sequencing bioinformatic workflow

Bulk RNA-sequencing data was used to produce heatmaps of known podocyte markers, pathway analysis using progeny and differential gene expression. Differential gene expression analysis was carried out using DESeq2 version 1.32.0 with internal statistical and normalization methods (i.e. multiple testing correction with Benjamini–Hochberg). The list of up- and downregulated genes that was obtained from DESeq2 for repaired podocytes versus mutant podocytes were used as input for enrichment analysis. Enrichment analysis for GO terms, KEGG pathways and Hallmark H gene sets was performed using clusterProfiler.

### RNAscope

Formalin-fixed paraffin-embedded (FFPE) kidney organoid samples were pretreated according to the protocol of ACDbio (322452, Bio-Techne). The probes that were used in this protocol were: RNASCOPE^®^ probe Hs-COL4A3 (461861, Bio-Techne); RNASCOPE^®^ probe Hs-PECAM1-O1-C2 (487381-C2, Bio-Techne); RNASCOPE^®^ probe Hs-NPHS1-C3 (416071-C3, Bio-Techne); and RNASCOPE^®^ 3-Plex negative control probe (320871, Bio-Techne). RNAscope was performed according to the manufacturer's protocol of ACDbio for fluorescent multiplex RNA *in situ* hybridization (320293-USM). Images were captured using a Zeiss LSM 880 confocal microscope.

### CRISPR/Cas NPHS2 repair (c.413G>A to c.413G)

CRISPR/Cas repair was performed by the Radboudumc Stem Cell Technology Center. In short, 70-90% confluent *NPHS2* mutant iPSCs were dissociated into single cells using TrypLE Select Enzyme for 2 min at 37°C. Cells were nucleofected with an RNP complex containing Alt-R^®^ S.p. Cas9 Nuclease V3 [1081058, Integrated DNA Technologies (IDT)] coupled to Alt-R^®^ S.p. CRISPR-Cas9 sgRNA 5′ AGAGTAATTATATTCCAACT 3′ (IDT) and Alt-R™ HDR donor oligo 5′ GGTGTTTAGAAAAAAAAGAGTGTTTTTTTACCAGGGCCTTTGGCTCTTCCAGGAAGCAGATGTCCGAGTCGGAATATAATAACCCTTTCATACTCTTG 3′ (PS modification, antisense; IDT) using a 4D-nucleofector System X Unit (Lonza) and the P3 primary Cell 4D-Nucleofector^®^ X kit (V4XP-3024, Lonza). Nucleofected cells were seeded in Matrigel matrix-coated (1:50, Corning) 10 cm dishes and cultured in E8 medium supplemented with Alt-R^®^ HDR Enhancer (1081072, IDT) and E8 supplement for 24 h. On day 2, medium was refreshed to E8 medium with E8 supplement and cells were cultured until colonies were observed. Multiple clones (∼25) were picked, frozen and made into a pellet for subsequent processing. After proteinase K treatment, DNA was extracted from these cell pellets and, following PCR and purification, Sanger sequencing was performed to confirm successful gene editing. Clones were screened for correct gene editing using Chromas Lite and ICE analysis software (Synthego). For sequencing data, including the repaired mutation, see Table S4.

### Fiji (ImageJ) quantification

Fluorescence images obtained from protamine sulfate experiments were analyzed using custom-made macros in the free open-software Fiji/ImageJ (see Zenodo, https://doi.org/10.5281/zenodo.5156000). First, the region of interest (ROI), was automatically determined by selection of the NPHS1^+^ area (podocytes) using a readily available Fiji threshold algorithm. The same area selection algorithm was used for all raw images. Second, mean phalloidin intensity was measured in NPHS1^+^ areas to automatically determine changes in cytoskeleton arrangement (cytoplasmic contraction). As an internal quality control for automatic threshold selection, images containing the respective ROI selection were automatically created for post-processing analysis to ensure correct ROI selection in all conditions.

### Statistical analysis

All data are expressed as mean±s.d. of three independent experiments, unless stated otherwise. Statistical analysis was performed using one-way ANOVA analysis followed by Tukey post-test or, when appropriate, an unpaired *t*-test with GraphPad Prism version 9. One asterisk was used to indicate significance with *P*<0.05, whereas two asterisks were used to indicate significance with *P*<0.01.

## Supplementary Material

Supplementary information
