# Supplementary material for: Human pluripotent stem cell-derived kidney organoids for personalized congenital and idiopathic nephrotic syndrome modeling
Source: Development. 2022 May 6;149(9):dev200198. doi: 10.1242/dev.200198 (PMC9148570; doi:10.1242/dev.200198)
Supplement: Supplementary information [file develop-149-200198-s1.pdf]

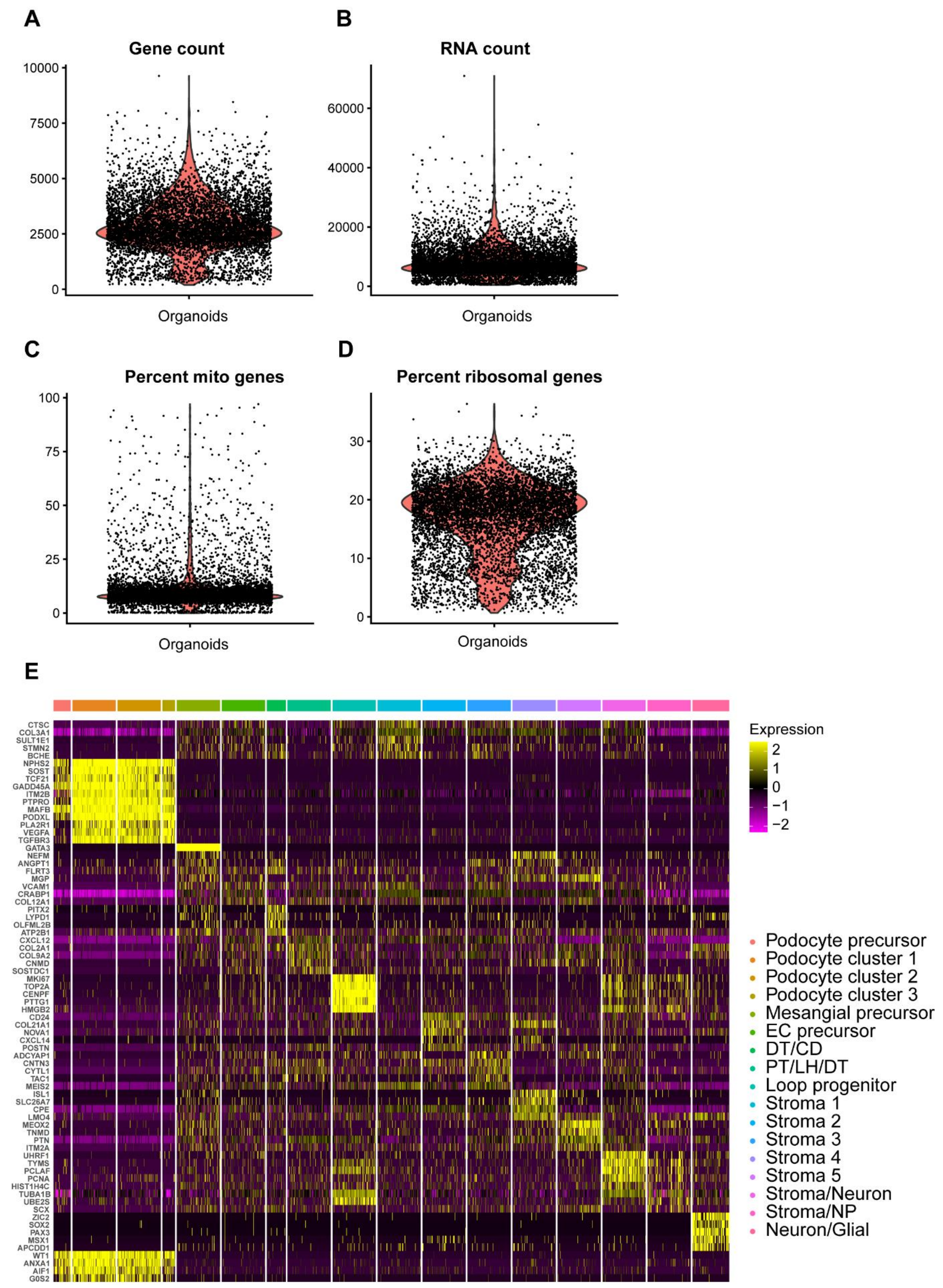

Fig. S1. Related to figure 1. Quality control data of scRNA seq data. (A) Number of genes, (B) total number of RNA molecules, percentage of (C) mitochondrial and (D) ribosomal genes in the organoids sample. (E) Heatmap showing a selection of 5 out of the top 20 differentially expressed (DE) genes per cluster. The full list of the top 20 DE genes per cluster is available in Supplemental Table S1. PT: proximal tubule, LH: loop of Henle, DT: distal tubule, CD: collecting duct, EC: endothelial cells.

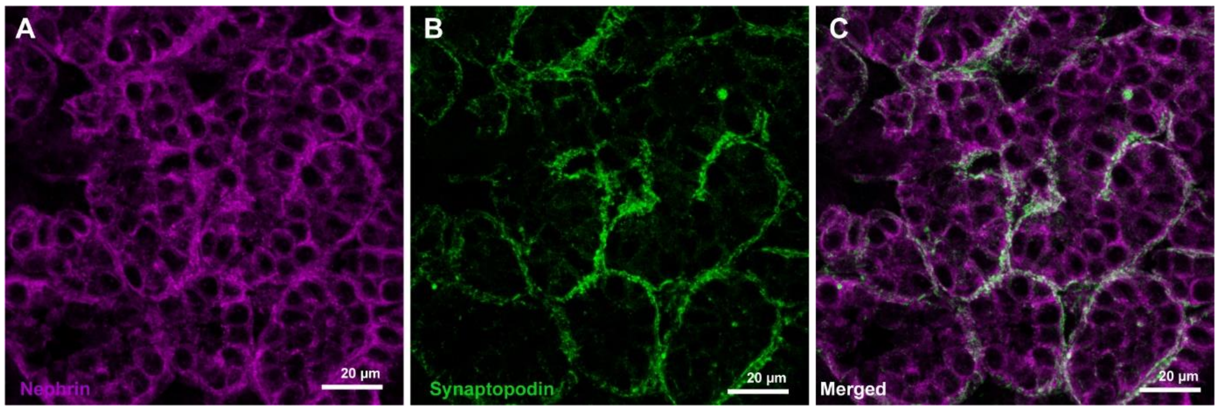

Fig. S2. Related to figure 1. Protein expression and localization of specific podocyte markers in kidney organoids. (A) Nephrin (magenta) and (B) synaptopodin (green) expression in organoid podocytes (C, merged).

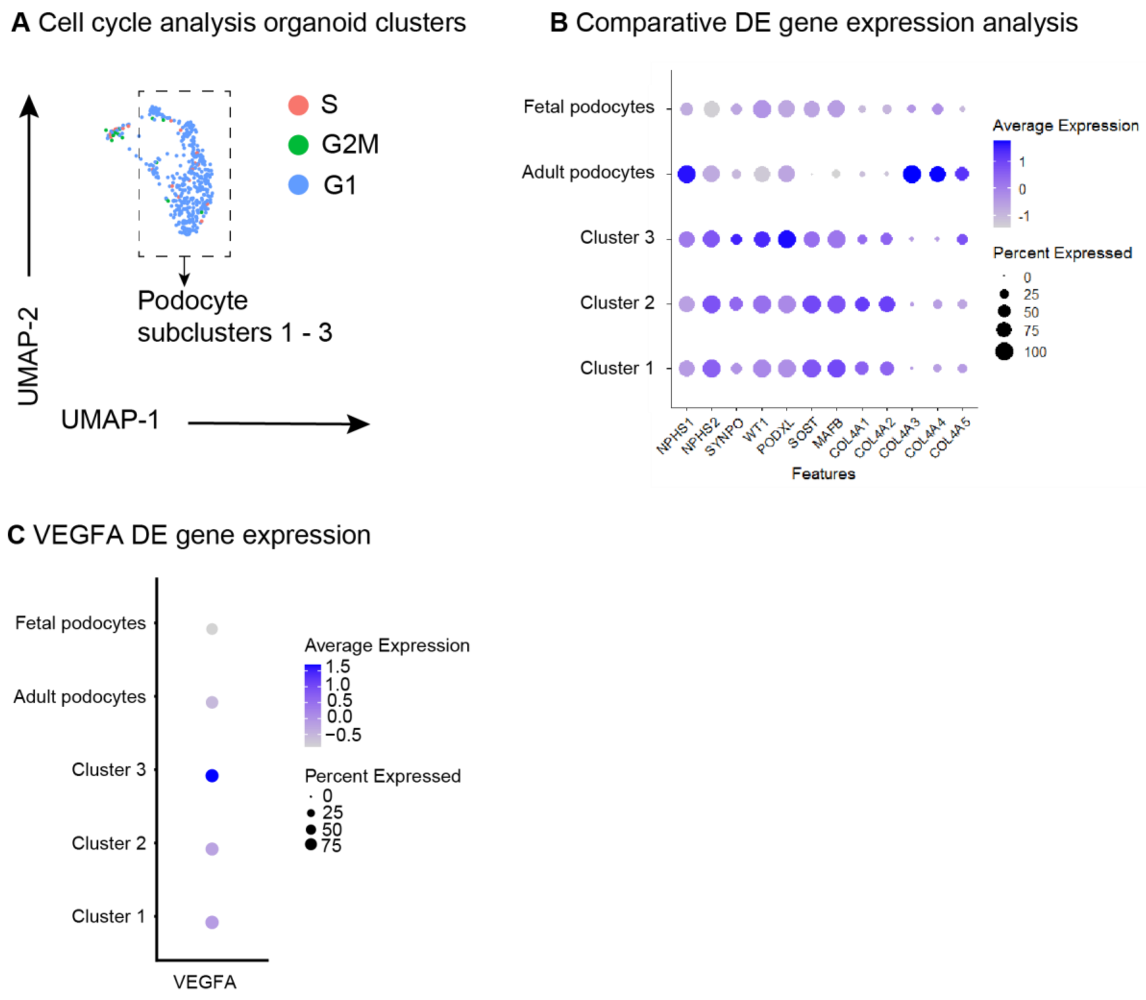

Fig. S3. Related to figure 2. Cell cycle analysis of the podocyte sub-clusters, comparative DE marker genes and comparative VEGFA expression of organoid podocyte sub-clusters, human fetal and adult podocytes. DE: differentially expressed.

Development • Supplementary information

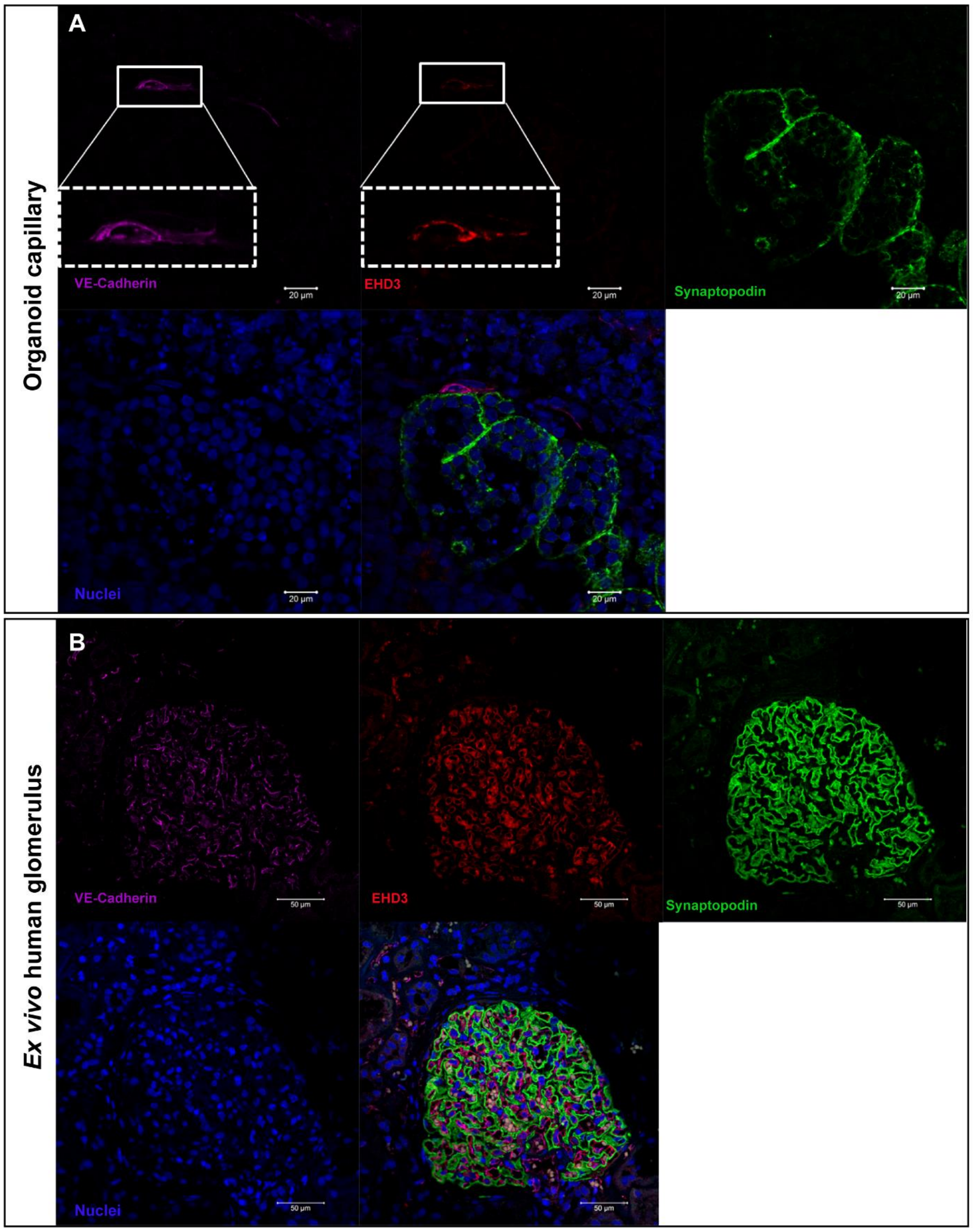

Fig. S4. Related to figure 2. Glomerular endothelial EHD3 expression in organoids and in human glomeruli. (A) VE-cadherin (magenta) and EHD3 (red) expression co-localized in an endothelial capillary in a kidney organoid, adjacent to synaptopodin expressing (green) podocytes. (B) VE-cadherin (magenta) and Eps15 Homology Domain-containing 3 (EHD3, red) expression co-localized in glomerular endothelium EHD3, in close proximity to podocytes as shown by synaptopodin expression.

Development • Supplementary information

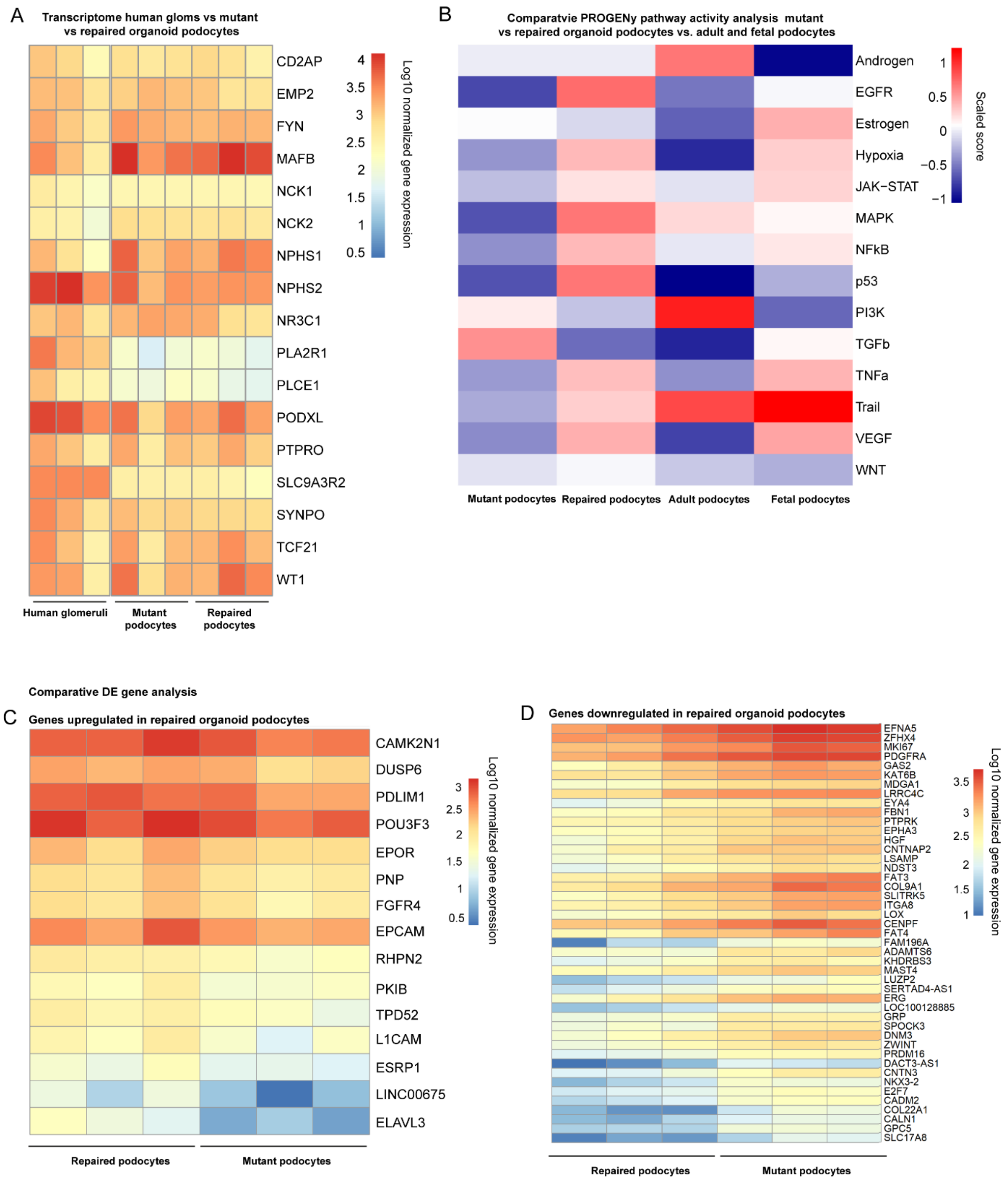

Fig. S5. Related to figure 4. Comparative RNA sequencing analysis of kidney organoid *NPHS2* mutant podocytes, repaired organoid podocytes and normal human glomeruli-derived podocytes. (A) Comparative bulk transcriptomics of podocyte specific genes in mutant and repaired organoid podocytes versus isolated human glomeruli. (B) Comparative PROGENy pathway activity analysis mutant and repaired organoid podocytes versus adult and fetal podocytes. Heatmap showing the enriched up- (C) and downregulated (D) genes in NPHS2-repaired organoid-derived podocytes compared to mutant organoid-derived podocytes.

Development • Supplementary information

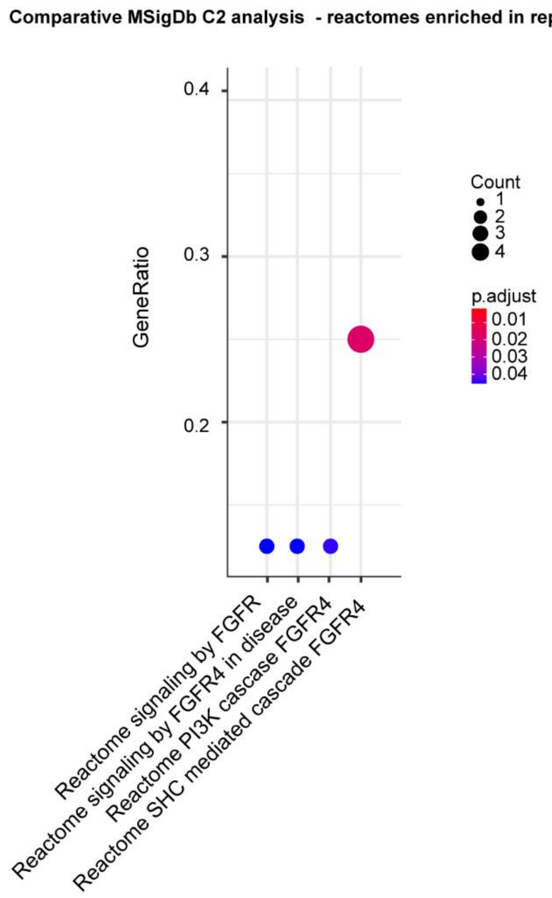

Fig. S6. Related to figure 4. Comparative Molecular Signature database Analysis C2 showing enhanced FGFR reactomes in repaired organoid podocytes.

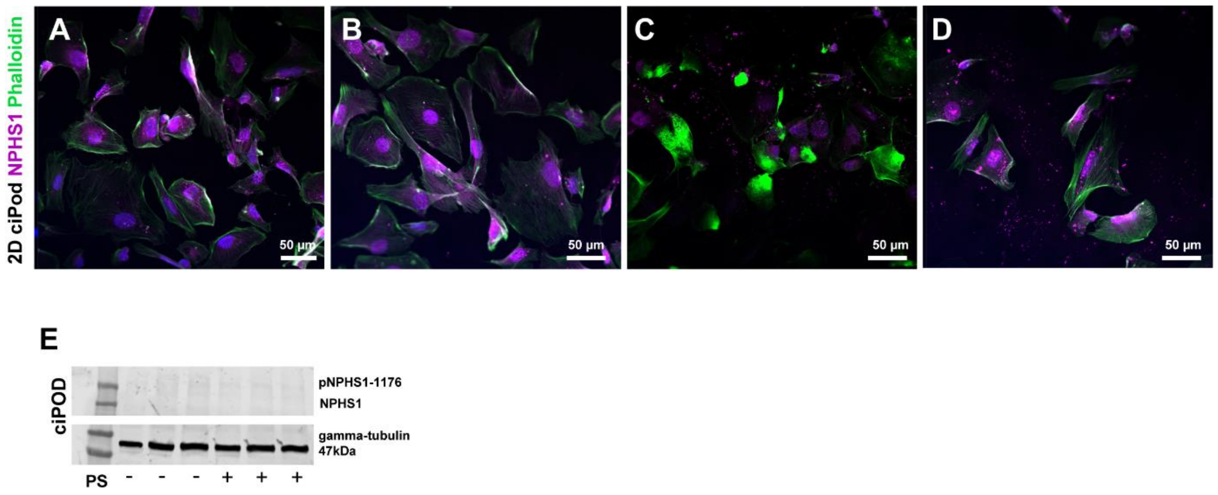

Fig. S7. Related to figure 5. Podocyte cytoskeleton analysis and slit diaphragm activation measurements in ciPODs following protamine sulfate treatment. (A-D) Phalloidin (green), NPHS1 (magenta)) of protamine sulfate treated and heparin rescued ciPODs. (E) pNPHS1 and NPHS1 protein expression analysis of protamine sulfate treated ciPODs. Gamma tubulin was used as a reference protein.

Development • Supplementary information

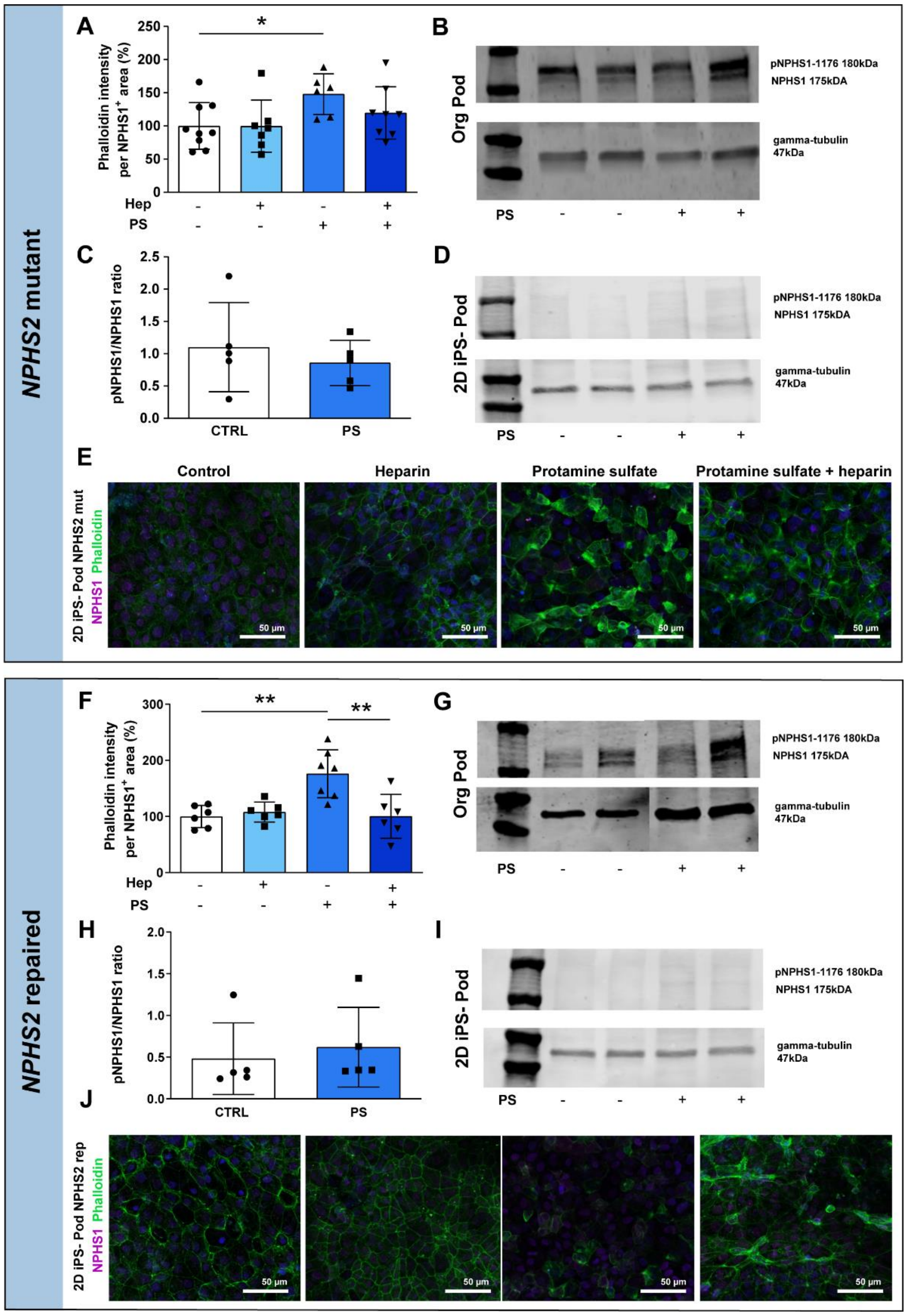

Development • Supplementary information

Fig. S8. Related to figure 5. Podocyte cytoskeleton analysis and slit diaphragm activation measurements in mutant and repaired kidney organoids. Quantification of phalloidin intensity in protamine sulfate-treated and heparin-rescued podocytes in (A) mutant and (F) repaired kidney organoids. *=p<0.05, ** = p<0.01, using one-way ANOVA analysis followed by Tukey post-test. Data are presented as mean ± SD from three independent experiments using at least two biological replicates per conditions. pNPHS1 and NPHS1 protein expression analysis of protamine sulfate-treated (B) mutant and (G) repaired kidney organoids. Western blotting quantification pNPHS1/NPHS1 ratio of protamine sulfate-treated (C) mutant and (H) repaired kidney organoids. Data are presented as mean ± SD from three independent experiments using at least two biological replicates per conditions. pNPHS1 and NPHS1 protein expression analysis of protamine sulfate-treated 2D (D) mutant and (I) repaired iPSC-derived podocytes. Gamma tubulin was used as a reference protein. Podocyte cytoskeleton analysis (phalloidin (green)) in (E) mutant and (J) repaired 2D iPSC-derived podocytes (NPHS1 (magenta)) following protamine sulfate treatment and heparin rescue.

Development • Supplementary information

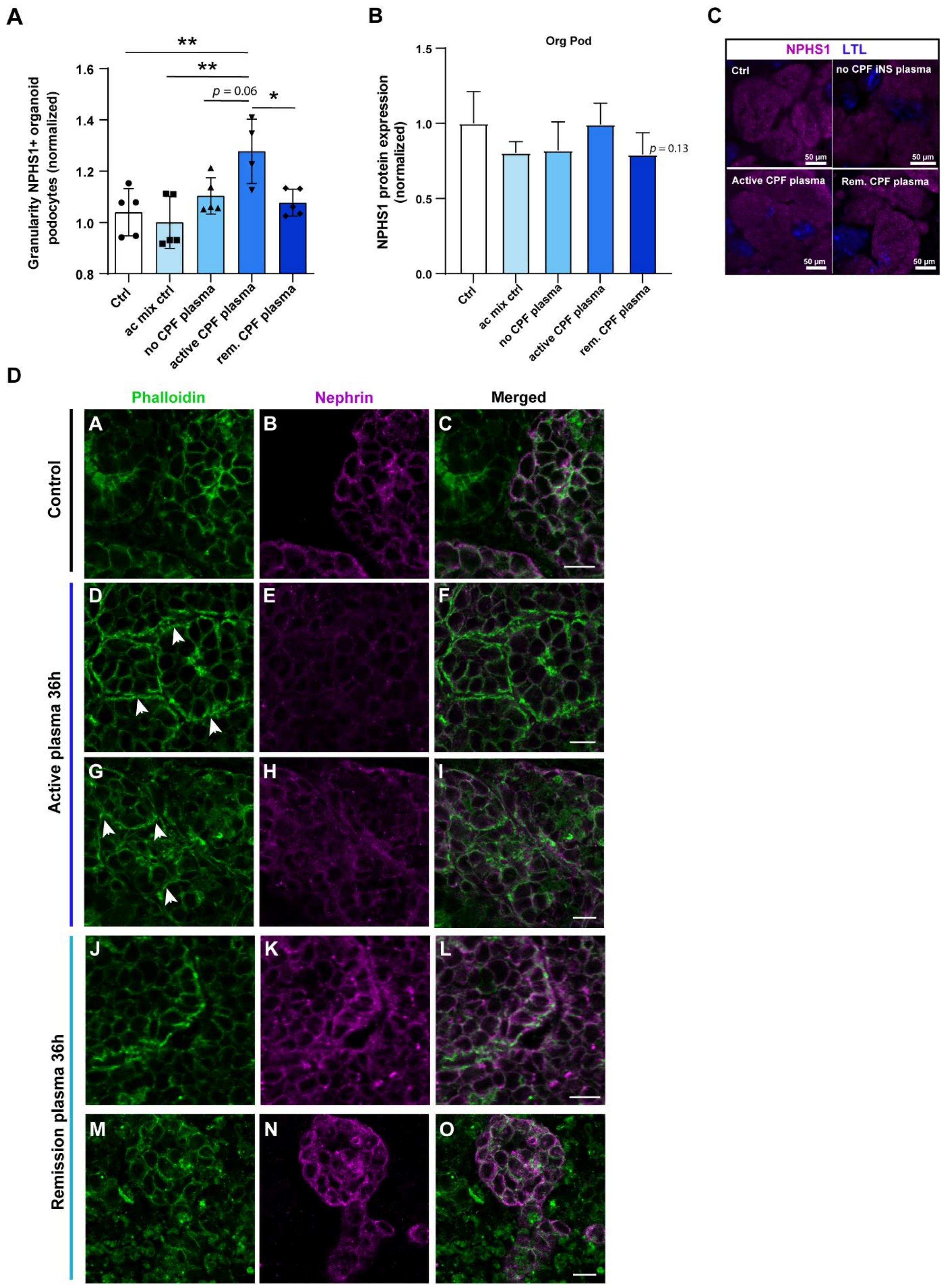

Fig. S9. Podocyte injury analysis following CPF-containing and -free plasma after 4h and 36h. (A) Cytoplasmic granularity analysis in NPHS1-sorted organoid podocytes, isolated from kidney organoids treated with 10% (v/v) iNS plasmapheresis plasma (circulating permeability factor (CPF) free, active CPF and remission CPF plasma), control (E6 medium) and anti-coagulation mix (ac mix ctrl) for 4h (N=2 independent experiments, biological triplicates per condition). *=p<0.05, **=p<0.01, using one-way ANOVA analysis followed by Tukey post-test. Data are presented as mean ± SD. (B) Flow cytometry analysis of NPHS1 expression in organoid podocytes (Org Pod) treated with 10% (v/v) active and remission CPF plasma, CPF-free plasma, anticoagulation control (Ac mix ctrl) and E6 medium control (Control) for 4h. Data are presented as mean ± SD. (C) NPHS1 (magenta) and LTL (blue) expression following 4h plasma treatments in kidney organoids. (D) Podocyte cytoskeleton analysis following CPF-containing (active) and -free (remission) plasma after 36h exposure. Arrowheads indicate actin condensation at the podocyte basement membrane. Scale bars 10 microns.

Development • Supplementary information

Table S1. Top 20 differentially expressed (DE) genes based on adjusted p-value

	PT/LH/DT	Podocyte precursor	Podocyte subcluster 1	Podocyte subcluster 2	Podocyte subcluster 3	Loop progenitor	DT/CD	EC precursor	Mesengial precursor	Stroma 1	Stroma 2	Stroma 3	Stroma 4	Stroma 5	Stroma/neural progenitor	Stroma/ neuron	Neuron/ glial
1	IRX1	WT1	NPHS2	CLIC5	ITGA3	KIF20A	LBX1	CD34	GATA3	CTSC	CD24	ADCYAP1	ISL1	MEOX2	CD81	CLSPN	ZIC2
2	IRX2	MAFB	SOST	PTPRO	PTPRO	DLGAP5	PITX2	PLVAP	NEFM	COL3A1	ALX4	CYTL1	OSR2	DLX2	COL3A1	XRCC2	SOX2
3	MAB21L2	GADD45A	TCF21	SOST	PODXL	CENPA	POU4F1	EBF3	ANGPT1	SULT1E1	PRRX1	CNTN3	NEFM	DLX1	COL1A2	TYMS	PAX3
4	RFLNA	NPHS2	PODXL	WT1	NPHS1	CENPE	TMEM108	VCAM1	FLRT3	CRABP1	COL21A1	PRSS12	SIM2	RALYL	MT-ND1	UHRF1	RFX4
5	CLDN11	MDK	PTPRO	SBSPON	PLA2R1	KNL1	GATA3	CRABP1	VCAN	CHST2	NOVA1	TAC1	ZNF385D	PALMD	HNF1B	CENPU	CACNG8
6	MECOM	MT-CO2	CLIC5	PODXL	ENPEP	CDC20	GRIA4	IFITM3	FN1	NAV3	CXCL14	VEGFC	HAND2	FSTL5	LAPTM4A	DTL	POU3F2
7	CXCL12	MARCKS	WT1	NPHS2	ST3GAL6	GTSE1	MYF6	COL12A1	PDGFC	STMN2	MAB21L2	CRABP1	SLC26A7	MGP	SFRP2	FAM111B	ZIC5
8	PTN	FTH1	DDN	NPHS1	NPHS2	HMMR	LYPD1	COL6A3	PTH1R	S100A10	MAB21L1	MEIS2	CHRM2	TNMD	HSP90B1	ORC6	CHL1
9	SIX2	MT-ATP6	AQP3	AQP3	AC008264.2	TACC3	CAMK2N1	LAMA4	FHL1	NDST3	ALDH1A2	TMSB4X	TTN	IFI44L	GAS1	RAD51AP1	SMOC1
10	USH1C	KCNQ1OT1	ENPEP	TCF21	CLIC5	PLK1	GATA3-AS1	CD99	AC060834.2	PENK	POSTN	FAT4	GUCY1A1	HPGD	FBLN1	MYBL2	MSX1
11	COL2A1	MT-CO3	SBSPON	DDN	SPOCK2	NUF2	OLFML2B	PCOLCE	TNMD	MEIS2	ALX1	CDH2	CPE	TRPS1	PDGFRA	PCLAF	PHYHIPL
12	COL9A2	SOX4	PLA2R1	VAMP8	SBSPON	ASPM	PDGFC	HMGA2	MEOX2	BCHE	CSRP2	MIR137HG	NRP2	MKX	MT-CYB	GINS2	PI15
13	EGFL6	MT-ND4	ST3GAL6	ST3GAL6	SOST	CCNB2	LIN7A	USH1C	TMSB4X	ACKR3	LVRN	SPOCK3	TBX2	COL11A1	CPE	ATAD5	MAP6
14	ERG	MT-ND1	SPOCK2	ENPEP	VEGFA	CKAP2L	SNCA	DDR2	FAM110B	TPM1	FAM196A	USH1C	SORCS1	FIBIN	MT-CO3	E2F1	LMX1A
15	PLD5	MT-CO1	VAMP8	MPP5	WT1	KIF4A	FLRT3	BTBD3	BCHE	TWIST1	LUM	RPL3	CCDC141	CSMD3	MDK	MCM10	TFAP2B
16	TSPAN13	CRABP1	CLDN5	CLDN5	PRODH2	TROAP	SHISA2	SERPINH1	MDK	COL6A3	PRRX2	RPL10	NRXN1	C3orf80	CALR	CDC45	GDF7
17	MIA	ANXA1	NPHS1	SPOCK2	MPP5	TOP2A	PTPRZ1	ROBO2	NR2F1	KLHL4	SOX11	RPL13	IGFBP3	RGCC	MT-ATP6	CENPK	RAB3C
18	CYP1B1	AIF1	MRGPRF	ARHGAP29	PTPRQ	MKI67	EGFLAM	TPM1	KIF26B	PARM1	TBX3	HGF	COL21A1	THBS4	IL11RA	CDC6	SATB2
19	SOX5	TPM2	STON2	PLA2R1	PLTP	KIF2C	CRABP1	CHST2	ADAMTS6	SLIT2	PCDH9	RPL10A	SIM1	RUNX1T1	FSTL1	MCM4	TTYH1
20	FAT3	COL3A1	TPPP3	GADD45A	MAGI2	BIRC5	ADAMTS6	EDNRA	CREB5	MAB21L2	PCDH10	RPL30	PDZRN3	PTGFR	APP	FANCD2	WSCD2

PT: proximal tubule, LH: loop of Henle, DT: distal tubule, CD: collecting duct, EC: endothelial cells.

Development • Supplementary information

Table S2. Antibodies used for immunofluorescence staining.

**Primary antibody**	**Secondary antibody**
Human Nephrin Antibody (AF4269, R&D Systems)	Donkey anti-sheep Alexa Fluor™ 647 (A21448, Thermo Fisher)
Anti-Podocin antibody (P0372, Sigma Aldrich)	Donkey anti-rabbit Alexa Fluor™ 568 (A10042, Thermo Fisher)
Anti-Human WT1 clone 6F-H2 (M3561, Dako)	Donkey anti-mouse Alexa Fluor™ 488 (A21202, Thermo Fisher)
Anti-synaptopodin antibody (65194, Progen)	Donkey anti-mouse Alexa Fluor™ 488 (A21202, Thermo Fisher)
Monoclonal Anti-PLA2R antibody (AMAB90772, Sigma Aldrich)	Donkey anti-mouse Alexa Fluor™ 488 (A21202, Thermo Fisher)
Human CD31/PECAM-1 Antibody (BBA7, R&D Systems)	Donkey anti-mouse Alexa Fluor™ 488 (A21202, Thermo Fisher)
PV-1 (PLVAP) antibody (HPA002279, Sigma-Aldrich)	Donkey anti-rabbit Alexa Fluor™ 568 (A10042, Thermo Fisher)
VE-cadherin (F-8) AC (sc-9989 AC, Santa Cruz Biotechnology)	Donkey anti-mouse Alexa Fluor™ 647 (A31571, Thermo Fisher)
Lotus Tetragonolobus Lectin (LTL) biotinylated (B-1325, Vector Laboratories)	Streptavidin, Alexa Fluor™ 405 conjugate (S32351, Thermo Fisher)
Anti-E-Cadherin clone 36 (610181, BD Biosciences)	Donkey anti-mouse Alexa Fluor™ 488 (A21202, Thermo Fisher)
GATA-3 (D13C9) monoclonal Antibody (5852, Cell Signaling)	Donkey anti-rabbit Alexa Fluor™ 647 (A31573, Thermo Fisher)
Flash Phalloidin™ Green 488 (424201, Biolegend)	n.a.
Anti-EHD3 antibody (HPA049890, Sigma-Aldrich)	Donkey anti-rabbit Alexa Fluor™ 568 (A10042, Thermo Fisher)

Table S3. Antibodies used for western blotting.

**Primary antibody**	**Secondary antibody**
Anti-Nephrin (phosphor Y1176 + Y1193) Antibody (ab80299, Abcam)	Goat anti-rabbit, Alexa Fluor™ 680 (A-21076, Thermo Fisher)
Anti-Nephrin antibody (sc-19000, Santa Cruz)	Donkey anti-goat, Alexa Fluor™ 680 (A-21084, Thermo Fisher)
Anti-γ-Tubulin antibody (T6557, Sigma)	Donkey anti-mouse, DyLight 800 (SA5-10172, Thermo Fisher)

Development • Supplementary information

Table S4. iPS-NPHS2 mutant CRISPR sequencing data

Sequencing data before CRISPR repair (NPHS2 exon 3):

ATCTAGAAAAAAAAAAAAATCCAATTGAATCATTTTGAAGCAGCCTCAGAAGAAATTGCACTCTGA
AACAAAACATCACAAGTGGTATTAAAATATACTCCCTCTTTCCATTAAATAGCTAATGAGCAATA
CATATAAAAGCTAGTGCAGAACTCACAGTAAAATATAAGATTTAATATGCCTTGATAAGATTAA
TTTAGGGAAAGTTGGCCATGGATTTTAGATAATCATAAGTCTTTAATCAAAATTCTGTCTATGGG
TTCAAAAATTAACATGGTTAATATACTTTTTCATTTCTGAAATTTTACACTTACTAAATATAGATT
TTGGAAACTTAAGTATTAATAGAAATTTTTTCCTGGTTCTCAAAACAAAAAATTTCTGATATCTA
GGATCATTCTTATGCCAAGGCCTTTTGAAGACTTTTTCTTTCTGGGAGTGATTTGAAAGGATTAA
ATTTCTCTTTAGGTTGTA*CAAGAGTATGAAAGAGTAATTATATTCC**A**ACTGGGACATCTGCTTCCTGGA*
*AGAGCCAAAGGCCCTGGTAAAAAAACACTCTTTTTTTTCTAAACAC*CTCTCTCCTGACTTGCCAATTTC
TTCAACCCATGCAGATTTGTAATATGGACCTCAGATTAAATGAAGTAACTTGATTCATGATATCT
GAATTTTCCAATCTGTTACTTATAGGTTATTCAAATATTCTTCAGAGACTATTACTACTAGGTCAT
AGGTAGCCAAGAGAGAGAATTGGTACAGAGAGCCCACATGCCAGGGCAAGGCTTGCTGGAATA
GCAAGTTAGCTTAGGACCAATGGCTGGGGACTGATTTGAGTACGATGTGTATATGGTCAGAGTC
CTGTGAATTCTCAGAGAAAAGCTGAGCTAGTTCCCACTCCAGGTGCTCAGATCCAATCATGAGA
AACAGGCAAAGTTCAGCATTCAAATAACAAGTTGCTCTTCAGTTATAGGGATTTAAAAAACATA
GATTAAGCATTTCTTGTGGCTCA

Sequencing data after CRISPR repair (NPHS2 exon 3):

ATCTAGAAAAAAAAAAAAATCCAATTGAATCATTTTGAAGCAGCCTCAGAAGAAATTGCACTCTGA
AACAAAACATCACAAGTGGTATTAAAATATACTCCCTCTTTCCATTAAATAGCTAATGAGCAATA
CATATAAAAGCTAGTGCAGAACTCACAGTAAAATATAAGATTTAATATGCCTTGATAAGATTAA
TTTAGGGAAAGTTGGCCATGGATTTTAGATAATCATAAGTCTTTAATCAAAATTCTGTCTATGGG
TTCAAAAATTAACATGGTTAATATACTTTTTCATTTCTGAAATTTTACACTTACTAAATATAGATT
TTGGAAACTTAAGTATTAATAGAAATTTTTTCCTGGTTCTCAAAACAAAAAATTTCTGATATCTA
GGATCATTCTTATGCCAAGGCCTTTTGAAGACTTTTTCTTTCTGGGAGTGATTTGAAAGGATTAA
ATTTCTCTTTAGGTTGTA*CAAGAGTATGAAAGGGTTATTATATTCC**G**ACTGGGACATCTGCTTCCTGGA*
*AGAGCCAAAGGCCCTGGTAAAAAAACACTCTTTTTTTTCTAAACAC*CTCTCTCCTGACTTGCCAATTTC
TTCAACCCATGCAGATTTGTAATATGGACCTCAGATTAAATGAAGTAACTTGATTCATGATATCT
GAATTTTCCAATCTGTTACTTATAGGTTATTCAAATATTCTTCAGAGACTATTACTACTAGGTCAT
AGGTAGCCAAGAGAGAGAATTGGTACAGAGAGCCCACATGCCAGGGCAAGGCTTGCTGGAATA
GCAAGTTAGCTTAGGACCAATGGCTGGGGACTGATTTGAGTACGATGTGTATATGGTCAGAGTC
CTGTGAATTCTCAGAGAAAAGCTGAGCTAGTTCCCACTCCAGGTGCTCAGATCCAATCATGAGA
AACAGGCAAAGTTCAGCATTCAAATAACAAGTTGCTCTTCAGTTATAGGGATTTAAAAAACATA
GATTAAGCATTTCTTGTGGCTCA

RED = sgRNA, *Cursive = Homology Directed Repair (HDR) template*, **BOLD = mutation (edit)**

The HDR Template contains silent mutations in the protospacer adjacent motif (PAM) region in order to prevent re-cutting after successful editing.

Original sequence:
CAAGAGTATGAAAGAGTAATTATATTCCAACTGGGACATCTGCTTCCTGGAAGAGCCAAAGGCCCT
GGTAAAAAAACACTCTTTTTTTTCTAAACAC

Sequence after successful editing and incorporation of silent mutations:
CAAGAGTATGAAAGGGTTATTATATTCCGACTGGGACATCTGCTTCCTGGAAGAGCCAAAGGCCCT
GGTAAAAAAACACTCTTTTTTTTCTAAACAC

Table S5. Reagent and resource table

Click here to download Table S5

Development • Supplementary information

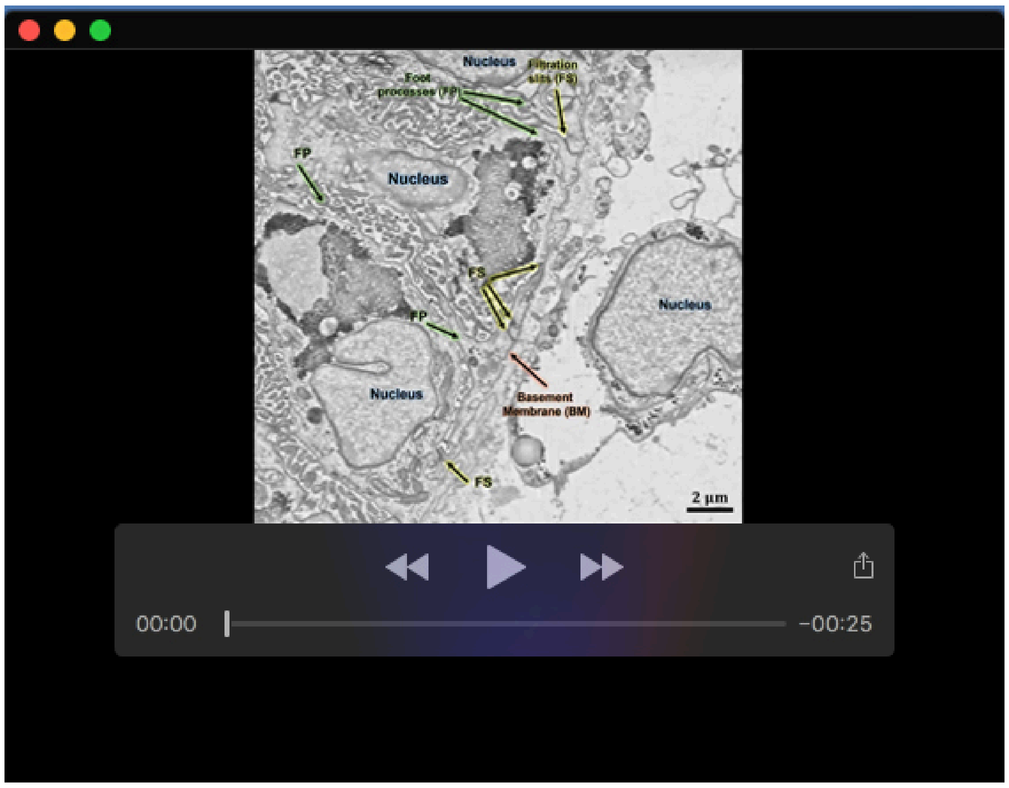

Movie 1. Related to figure 6. FIB-SEM volume imaging of the vehicle-treated organoid podocytes showing segmented podocyte cell bodies, foot processes and annotated filtration slits.

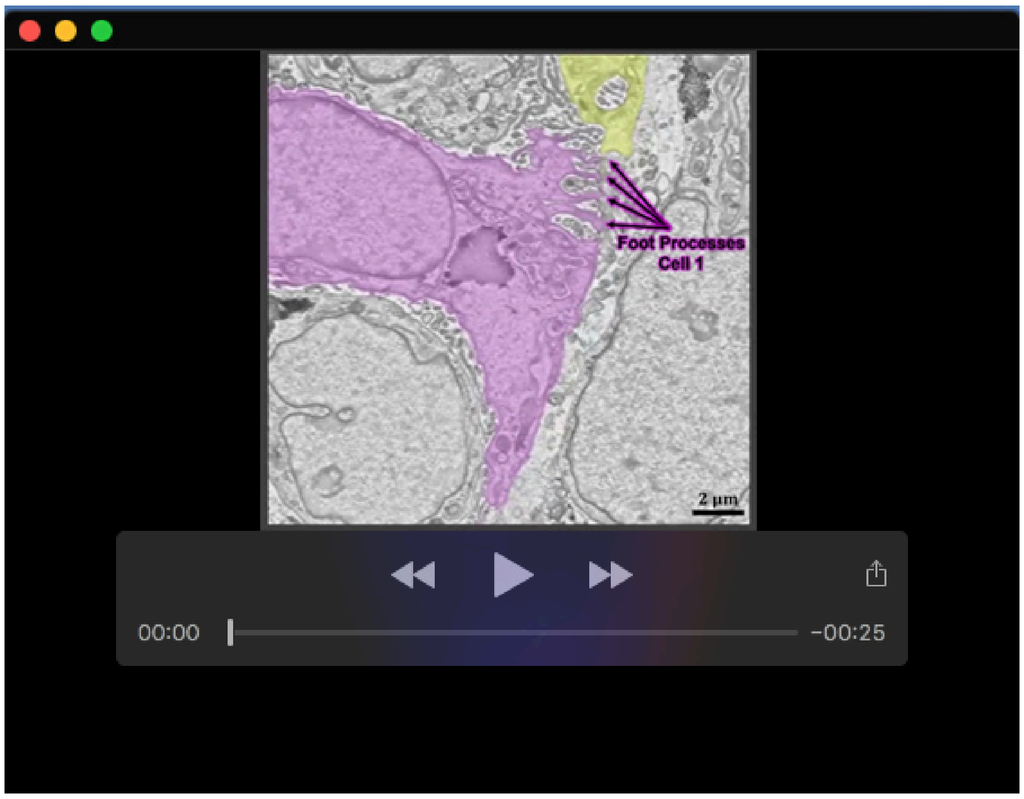

Movie 2. Related to figure 6. 3D rendering following FIB-SEM volume imaging and segmentation of the vehicle-treated organoid podocytes showing interdigitating foot processes.

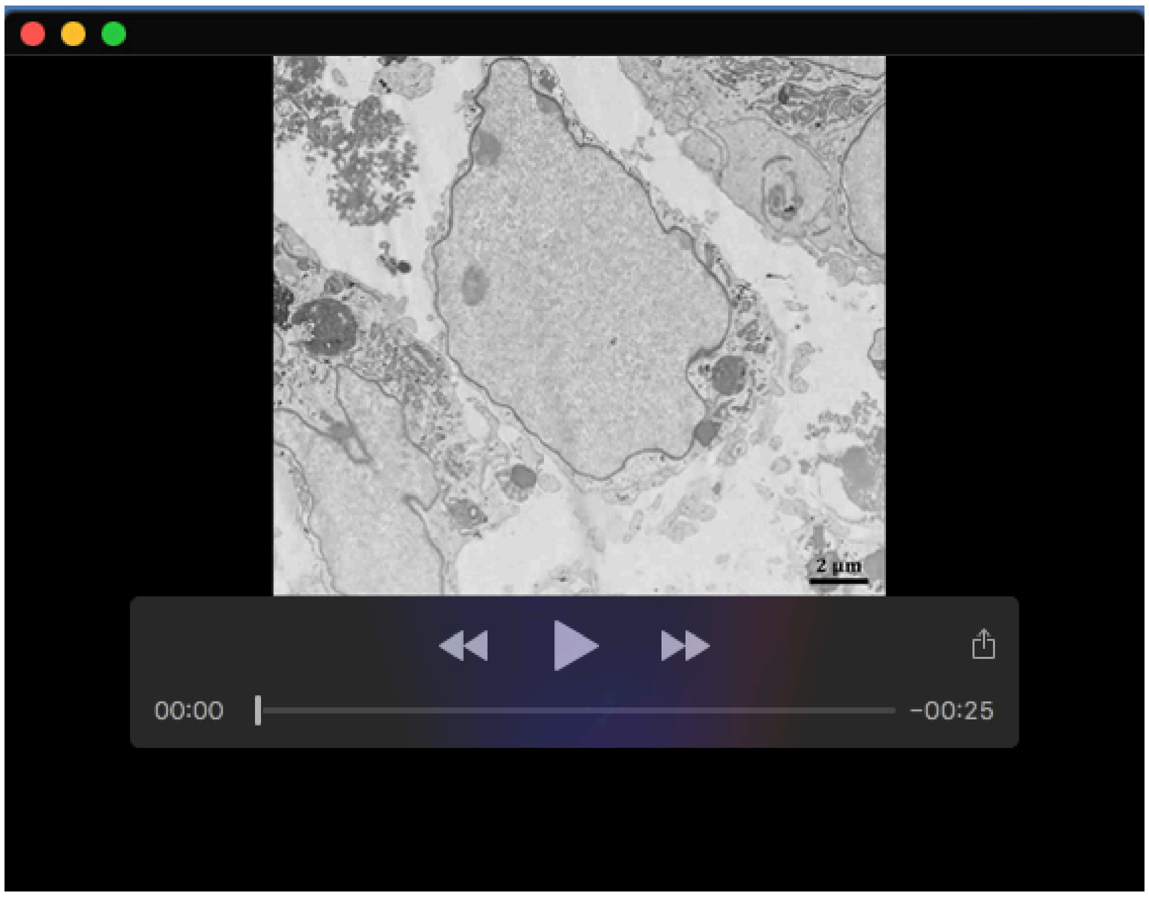

Movie 3. Related to figure 6. FIB-SEM volume imaging of the PAN-treated organoids showing injured podocytes.
